# Dual inhibition of IDO1/TDO2 enhances anti-tumor immunity in platinum-resistant non-small cell lung cancer

**DOI:** 10.1186/s40170-023-00307-1

**Published:** 2023-05-24

**Authors:** Chunjing Wu, Sydney A. Spector, George Theodoropoulos, Dan J. M. Nguyen, Emily Y. Kim, Ashley Garcia, Niramol Savaraj, Diane C. Lim, Ankita Paul, Lynn G. Feun, Michael Bickerdike, Medhi Wangpaichitr

**Affiliations:** 1 Department of Veterans Affairs, Miami VA Healthcare System, Miami, FL USA; 2 Department of Medicine, University of Miami School of Medicine, Miami, FL USA; 3 Department of Electrical and Computer Engineering, Drexel University, Philadelphia, PA USA; 4 Antido Therapeutics Pty Ltd, Melbourne, Australia; 5 Department of Surgery, University of Miami School of Medicine, Miami, FL USA

**Keywords:** Lung cancer, Kynurenine, Dual IDO1/TDO2 inhibitors, Cisplatin resistance, Immunometabolism, Drug resistance

## Abstract

**Background:**

The impact of non-small cell lung cancer (NSCLC) metabolism on the immune microenvironment is not well understood within platinum resistance. We have identified crucial metabolic differences between cisplatin-resistant (CR) and cisplatin-sensitive (CS) NSCLC cells with elevated indoleamine 2,3-dioxygenase-1 (IDO1) activity in CR, recognized by increased kynurenine (KYN) production.

**Methods:**

Co-culture, syngeneic, and humanize mice models were utilized. C57BL/6 mice were inoculated with either Lewis lung carcinoma mouse cells (LLC) or their platinum-resistant counterpart (LLC-CR) cells. Humanized mice were inoculated with either A (human CS cells) or ALC (human CR cells). Mice were treated with either IDO1 inhibitor or TDO2 (tryptophan 2,3-dioxygenase-2) inhibitor at 200 mg/kg P.O. once a day for 15 days; or with a new-in-class, IDO1/TDO2 dual inhibitor AT-0174 at 170 mg/kg P.O. once a day for 15 days with and without anti-PD1 antibody (10 mg/kg, every 3 days). Immune profiles and KYN and tryptophan (TRP) production were evaluated.

**Results:**

CR tumors exhibited a more highly immunosuppressive environment that debilitated robust anti-tumor immune responses. IDO1-mediated KYN production from CR cells suppressed NKG2D on immune effector natural killer (NK) and CD8^+^ T cells and enhanced immunosuppressive populations of regulatory T cells (Tregs) and myeloid-derived suppressor cells (MDSCs). Importantly, while selective IDO1 inhibition attenuated CR tumor growth, it concomitantly upregulated the TDO2 enzyme. To overcome the compensatory induction of TDO2 activity, we employed the IDO1/TDO2 dual inhibitor, AT-0174. Dual inhibition of IDO1/TDO2 in CR mice suppressed tumor growth to a greater degree than IDO1 inhibition alone. Significant enhancement in NKG2D frequency on NK and CD8^+^ T cells and a reduction in Tregs and MDSCs were observed following AT-1074 treatment. PD-L1 (programmed death-ligand-1) expression was increased in CR cells; therefore, we assessed dual inhibition + PD1 (programmed cell death protein-1) blocking and report profound anti-tumor growth and improved immunity in CR tumors which in turn extended overall survival in mice.

**Conclusion:**

Our study reports the presence of platinum-resistant lung tumors that utilize both IDO1/TDO2 enzymes for survival, and to escape immune surveillance as a consequence of KYN metabolites. We also report early in vivo data in support of the potential therapeutic efficacy of the dual IDO1/TDO2 inhibitor AT-0174 as a part of immuno-therapeutic treatment that disrupts tumor metabolism and enhances anti-tumor immunity.

**Supplementary Information:**

The online version contains supplementary material available at 10.1186/s40170-023-00307-1.

## Introduction

**Fig. 1:**
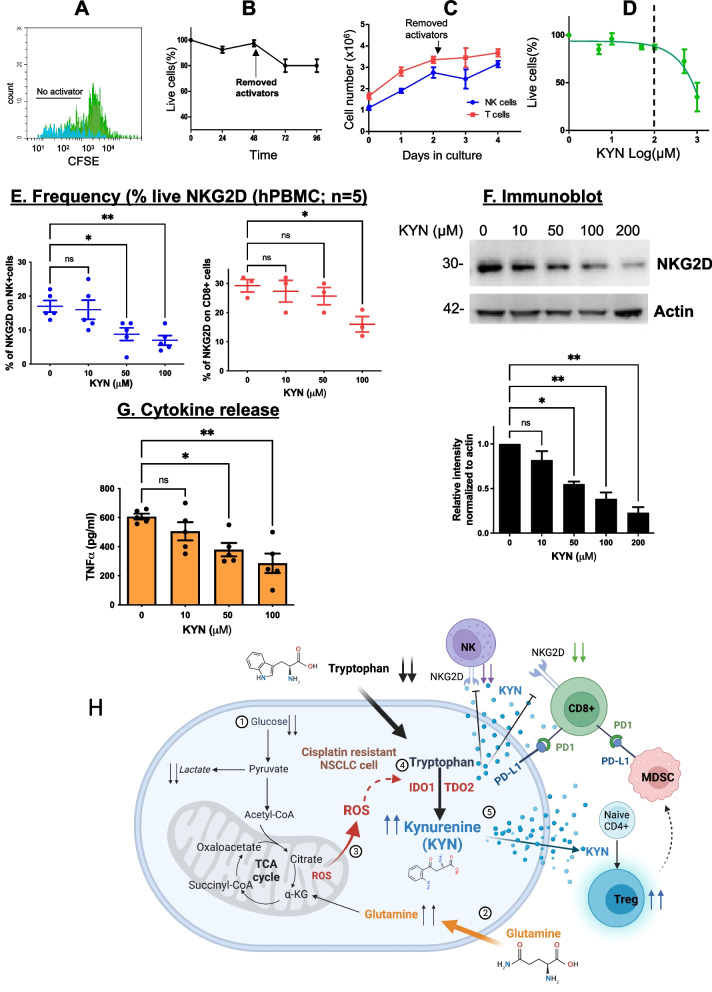
Increased KYN suppressed NKG2D expression of NK and CD8 + T cells. **A** Human PBMCs (hPBMCs) were cultured in the presence of an activator (CD2/28 + IL2) for 48 h. On day 3, cells were washed and the proliferation rate of lymphocytes was determined by assessing the reduction of the intensity of the fluorescent cell-permeable dye CFSE. **B** Growth inhibitory effect: PBMCs were exposed to activators, washed, and assayed for cell viability at each time point. **C** Results of NK + and CD8 + T cell expansion at each time point before and after the removing activator. **D** After activators were removed, activated hPBMCs were exposed to KYN at the indicated concentrations for another 48 h. We observed cytotoxic effects above 100 μM. **E** Using the same experimental conditions as 1D, the percent of NKG2D on NK + and CD8 + T cells were evaluated after KYN treatment. **F** Immunoblot of NKG2D from activated hPBMCs treated with increasing concentrations of KYN for 48 h. **G** Pro-inflammatory cytokine TNFα was quantified by the LEGENDplex™ bead-based immunoassay. **H** Immunometabolism scheme in cisplatin-resistant (CR) NSCLC cells. *(1)* CR lung cancer cells uptake less glucose and are no longer addicted to glycolysis compared to sensitive cells. *(2)* CR cells utilize amino acids (i.e., glutamine) as their main carbon skeleton source to survive. *(3)* Increased oxidative metabolism (OXMET) leads to increased accumulation of intracellular ROS. *(4)* ROS activates the KYN pathway via a ROS-dependent enzyme (IDO1) resulting in significant TRP uptake and greater KYN production. *(5)* Increased extracellular KYN activates Treg and suppresses NKG2D. CR cells with high IDO1 activity recruit and activate MDSCs via Treg [15]. PDL1 expression by CR cells and MDSCs further inhibits immune effector populations. In all the experiments, data are presented as mean ± SEM of 3 independent experiments and were analyzed using one-way ANOVA followed by Dunnett’s multiple comparisons with *P < 0.05, **P < 0.005, ***P < *0.0005*)

The effectiveness of platinum-based chemotherapies for non-small cell lung cancer (NSCLC) is limited by tumor mechanisms of drug resistance. While there are many mechanisms of resistance [1–3], we discovered that cisplatin-resistant (CR) NSCLC cells do not follow classic aerobic glycolysis (Warburg Effect) [4–6]. In addition, we found that CR cells, compared to cisplatin-sensitive (CS) cells, consume glutamine at a higher rate and generate an excessive level of reactive oxygen species (ROS) which activates the kynurenine (KYN) pathway [7]. Indoleamine 2,3-dioxygenase-1 (IDO1) is a ROS-scavenging enzyme that catabolizes tryptophan (TRP) to KYN [7, 8]. Recent studies showed that KYN and its immediate downstream metabolites (i.e., 3-hydroxy-kynurenine) are also potent inhibitors of the redox stress [9]. Other rate-limiting step enzymes involved in TRP degradation include tryptophan 2,3-dioxygenase-2 (TDO2) and IDO2 [10, 11]. We investigated this path further since increased KYN pathway (KP) activity has been described as one of the major mechanisms of immunosuppression in tumors [12–14] (Fig. 1H).

Although the mechanisms of KP have been studied for decades, the activity of this pathway remains poorly understood, and KP can lead to tumor growth and proliferation through several mechanisms. IDO1 enzyme can regulate immunosuppression in the tumor microenvironment (TME) by recruiting myeloid-derived suppressor cells (MDSCs) via the regulatory T cells (Tregs) [15]. In fact, immunosuppressive factors produced by MDSCs are also comprised of amino acid catabolizing enzymes (e.g., arginase-1 and IDO1) and immunosuppressive cytokines (e.g., IL-10 and TGF-β), leading to T cell exhaustion [16, 17]. The inhibition of IL-2 and anti-CD3/CD28 mAb-induced T cell amplification, and Th1 polarization are dependent on IDO1; thus, blocking IDO1 can induce immune effector cells and reverse MDSCs’ immunosuppressive activities [18]. As a metabolite, extracellular KYN is known to be involved in reprogramming naive T cells to Tregs (CD4^+^CD25^+^FoxP3^+^) thereby creating another layer of an immunosuppressive environment allowing cancers to evade the immune surveillance [19, 20]. To make matters worse, KYN also impairs natural killer cells (NKs) by preventing the upregulation of the natural killer group 2 member D (NKG2D) expression [21]. NKG2D is a type II transmembrane receptor that is important for the induction of NK-mediated elimination of target cells [22]. Signaling through NKG2D also results in a killing effect in CD8^+^ T cells which work along with T cell receptor activation to affect the function and promote immune responses [23]. It is important to note that studies of NSCLC derived from patients receiving several cycles of platinum-based therapy found downregulation of NKG2D ligands and upregulation of program death receptor ligand-1 (PD-L1), supporting the attenuation of NK and CD8^+^ T cell-mediated tumor cell death [24]. Consistent with this and other publications, we and others have shown that treatment with platinum-containing regimens resulted in higher expression of PD-L1 in solid tumors including NSCLC [25, 26]. PD-L1 expression in tumors is now frequently assessed for the implementation of checkpoint inhibitor therapy as a standard of care in patient management since it is a predictive marker for the efficacy of immune checkpoint inhibitor therapies [27]. Hence, the PD-L1/PD1 pathway is relevant in the description of the immune environments for CR tumors.

Compensatory changes in the signaling pathways of treated cancer cells leading to drug resistance is a well-known concept; hence, we considered it in our work. In this study, we found that inhibiting IDO1 induced upregulation of TDO2 protein both in vitro and in vivo. Therefore, we investigated the potential benefit of blocking both IDO1 and TDO2 in a syngeneic mouse model and a humanized mouse model of cisplatin-resistant NSCLC. We evaluated the effect of administering the orally bioavailable IDO1/TDO2 inhibitor AT-0174 alone and in combination with an anti-PD1 antibody on tumor growth and survival.

Our study demonstrates the impact of non-small cell lung cancer (NSCLC) tumor metabolism on immune cell profiles in the context of gaining a therapeutic advantage for modern therapeutic combination treatments that include immunotherapies and modulators of the immune response. This work provides important new in vivo evidence in support of a mutualistic role of IDO1/TDO2 in KYN-mediated cisplatin resistance in NSCLC, highlighting the therapeutic potential of dual IDO1/TDO2 inhibition in the treatment of NSCLC by reversing immunosuppressive conditions to active anti-tumor immune responsiveness.

## Results

### Increased extracellular kynurenine suppresses immune effector-NKG2D cells

In earlier reports, we observed increased populations of immunosuppressive cells in mice bearing CR tumors compared to CS tumors, where CR tumors had a higher Treg population and IDO1 activity measured by increased KYN concentrations [7]. Interestingly, a recent study reported that extracellular KYN inhibits the surface expression of NKG2D-activating receptors and regulates NK cell function [21]. To further assess the effect of KYN on immune effector cells, we first determined the receptor levels of NKG2D in response to different concentrations of exogenous KYN in vitro. We activated human peripheral blood mononuclear cells (hPBMCs) in RPMI media with anti-CD2/CD28 in combination with IL2. After 2 days of priming, we removed these activators and allowed cells to grow for another 48 h. Staining of residual cell proliferation dye (CFSE) indicated that PBMCs expanded and proliferated, thus demonstrating the system’s functionality and the survival of cells in these experimental conditions (Fig. 1A, B). Importantly, NK and CD8^+^ T cell populations continued to expand in the absence of activators, hence stimulation was ongoing (Fig. 1C). Using these conditions, stepwise increasing KYN concentrations were added to the culture with a media TRP concentration level of around 20 μM (human serum concentrations of tryptophan are in the range of 70–80 μM) [28]. Despite this caveat, growth inhibition was observed at KYN concentrations greater than 100 µM (Fig. 1D); thus, this concentration was the upper limit threshold for our experiments. Increasing the concentration of exogenous KYN significantly suppressed NKG2D expression on NK (CD3^−^CD56^+^), and CD8^+^ T (CD3^+^CD8^+^) cells (Fig. 1E; see the gating strategy in Supplemental Figure S1A). The decrease in NKG2D expression correlated with increasing KYN concentrations (Fig. 1F), and corresponded to a decrease in released pro-inflammatory cytokine TNFα (Fig. 1G). Consistent with other previous findings, our results confirm that increased KYN can suppress NKG2D expression on NK and CD8^+^ T cell populations, inhibiting immune activation.

### IDO1-mediated kynurenine production from cisplatin-resistant NSCLC Cells (CR) Suppresses immune effector cell populations

**Fig. 2:**
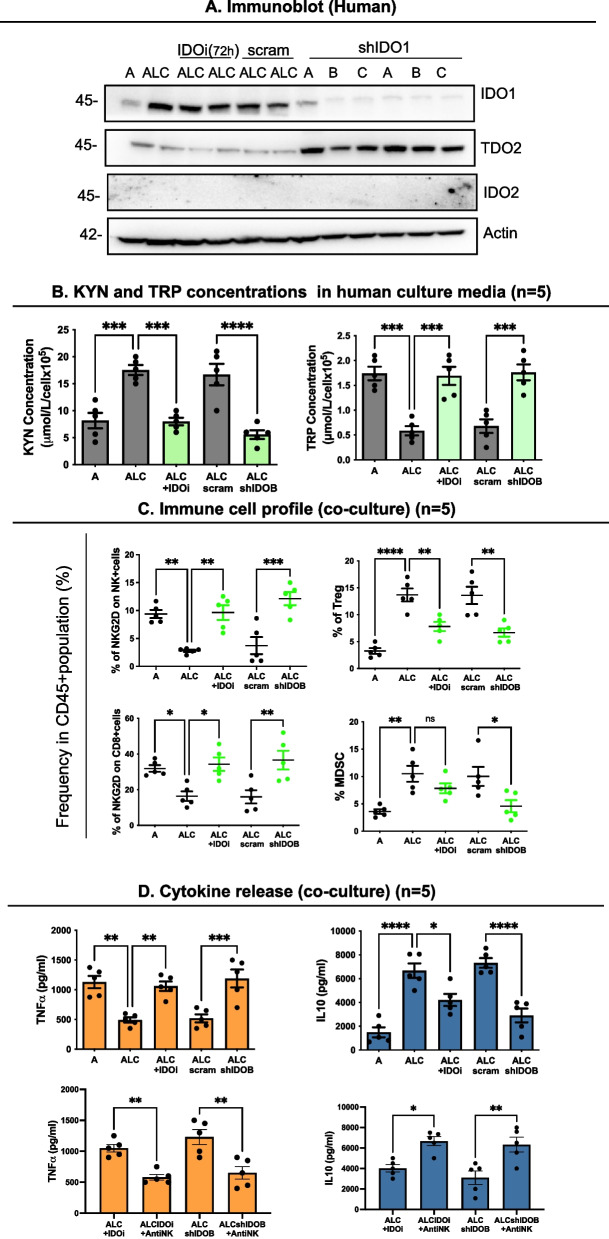
IDO1-mediated KYN production from CR cells suppressed immunomodulatory profile.** A** Immunoblot of IDO1, IDO2, and TDO2. Resistant cells were treated with either IDO1 inhibitor or shRNA targeting IDO1 (shCTRL represents scramble sequence, shIDO A, B, and C represent 3 unique shRNA sequences). **B** Detection of amino acid KYN and TRP concentrations in culture supernatants using Amino Acid Analyzer Biochrome30 + . **C** Immune profile of CS vs. CR co-cultured with hPBMC. Using the same experimental condition as Fig. 1E above, IDO1 inhibitions significantly enhanced the percent of NKG2D on NK cells (CD3-CD56 + NKG2D +) and the percent of NKG2D on CD8 + (CD3 + CD8 + NKG2D +) cells, but significantly suppressed Treg (CD4 + CD25 + FoxP3 +) and MDSC (HLA-DR.^lo^CD14-CD11b + CD33 +) populations (see the gating strategy in Supplemental Figure S1). **D** The indicated cytokines are quantified in culture supernatants by LEGENDplex™ bead-based immunoassay. The panel below indicated that anti-NKG2D blocking antibodies blunt the effect of IDO1 inhibition. Note: To detect the MDSC population, cells were activated by PHA + IL2 instead of antiCD2/28 + IL2. Cell lines A and FA are cisplatin-sensitive, and ALC and FC are cisplatin-resistant counterparts. In all experiments, data presented as mean ± SEM of 3 independent experiments and were analyzed using one-way ANOVA followed by Tukey’s multiple comparison analysis with *P < 0.05, **P < 0.005, ***P < 0.0005, ****P < *0.0001*

We have shown that CR cell metabolism involves higher IDO1 activity and KYN production when compared to CS cells [7]. Inhibition of IDO1 with knockdown (shIDO1) (Fig. 2A and Supplemental Figure S2A) or IDO1 inhibitor (IDOi) significantly suppressed KYN production, reversing TRP depletion in culture media, signifying the dependence of KYN production on IDO1 function in the CR model (Fig. 2B and Supplemental Figure S2B). To ascertain if increased KYN found in CR cells impacts NKG2D frequency on NK and CD8^+^ T cells, we established an in vitro co-culture system of human cancer cells (sensitive (A or FA) vs. resistant (ALC or FC)) with hPBMCs. Using transwell chambers, PBMCs were separated from cancer cells limiting their interaction to only soluble factors (Fig. 2C). Viable NK cells (CD3^−^CD56^+^NKG2D^+^), CD8^+^ T cells (CD3^+^CD8^+^NKG2D^+^), Tregs (CD4^+^CD25^+^FoxP3^+^), and MDSC (HLA-DR^lo^CD14-CD11b + CD33 +) populations were gated using flow cytometry (see gating in Supplemental Figure S1A). We found an increased percentage of NKG2D on NK and CD8^+^ T cells when IDO1 was inhibited. Importantly, CR cells induced higher Treg and MDSC populations compared to CS cell counterparts, but inhibition of IDO1 in CR cells markedly reversed these immunosuppressive cell populations (Fig. 2C and Supplemental Figure S2C). The resultant increase in immune effector populations upon IDO1 inhibition was paralleled by an increase in TNFα and decreased anti-inflammatory IL-10 cytokine production (Fig. 2D and Supplemental Figure S2D). Cytokine release was significantly reversed by blocking antibodies to NKG2D. Together, our data suggest that increased KYN levels from CR cells enhanced immunosuppressive cells, and hindered immune effector cells by modulating NKG2D receptor expression. Even though inhibiting the KYN pathway leads to pro-inflammatory environments in CR cells, the knockdown of IDO1 did not result in complete KYN inhibition. Thus, we anticipate that other rate-limiting step enzymes in the KYN-generating pathway may play a role in overcoming IDO1 inhibition in NSCLC.

### Role of dioxygenase enzymes in overcoming blockage of the kynurenine pathway

**Fig. 3:**
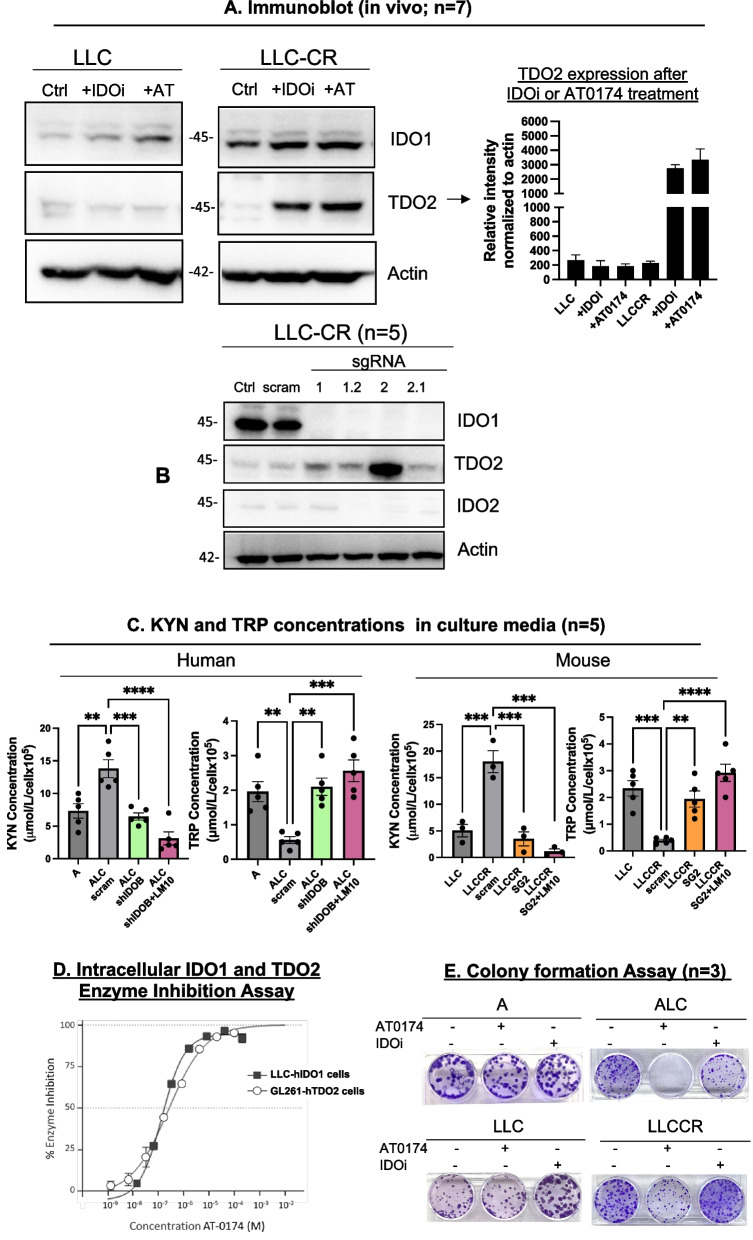
The compensatory role of IDO1 and TDO2 in cisplatin-resistant NSCLC. **A** Immunoblot of IDO1 and TDO2 from LLC vs. LLC-CR that were treated with either IDO1 inhibitor or dual inhibitor (AT-0174). The right panel indicated TDO2 relative intensity after IDO1 or IDO1/TDO2 blockage. **B** Immunoblot of IDO1, IDO2, and TDO2 expression in CRISPR-edited *Ido1* knockout SG1 and SG2 tumors. Scram indicated gRNA control. **C** KYN (amino acid) and TRP concentrations were detected in human and mouse cell cultures. Blocking both IDO1 and TDO2 led to a significant reduction in KYN secretions with higher extracellular TRP concentrations. **D** IDO1 and TDO2 enzyme inhibition in response to AT-0174. LLC-hIDO1 or GL261-hTDO2 cells were incubated with AT-0174 for 24 h. The supernatant was collected and assayed for kynurenine activity by colorimetric assay. The inhibition potency (IC_50_ value) of AT-0174 on the IDO1 and TDO2 enzymes were calculated to be 0.17 µM and 0.25 µM, respectively. **E** Colony formations of human and mouse cell lines were determined in CS (A or LLC) vs. CR (ALC or LLC-CR) cells treated with 25 µM of AT-0174 or IDOi for 3 days and reseeded for 12 more days. Cell lines A and LLC are cisplatin-sensitive, and ALC and LLC-CR are cisplatin-resistant counterparts. Data were analyzed using one-way ANOVA followed by Tukey’s multiple comparison analysis with *P < 0.05, **P < 0.005, ***P < *0.0005*

IDO2 and TDO2 are other enzymes with roles in KYN metabolism, where IDO2 is a very poor producer of KYN and has been suggested to function differently from IDO1 [29, 30]. TDO2 is generally known to only be expressed in the liver, kidney, brain, and with very low lung expression in normal tissue [31]. Consistent with these reports, we detected very low to non-detectable IDO2 and TDO2 proteins expressed in our CS and CR lung cancer cells as shown in Fig. 2A and Supplemental Figure S2A. However, IDO2 and TDO2 may be part of a compensatory/mutualistic mechanism induced when IDO1 is inhibited, providing another potential reason for the lack of efficacy presented in the phase 3 trial (ECHO-301) of epacadostat in combination with pembrolizumab for the treatment of melanoma [32]. Knocking down IDO1 led to a marked increase in TDO2 expression in CR cells (Fig. 2A and S2A) supporting the compensation concept. To determine whether inhibition of IDO1 may also induce IDO2 and TDO2 expression in vivo, C57BL/6 mice were inoculated with either Lewis lung carcinoma mouse cells (LLC) or its platinum-resistant counterpart (LLC-CR) cells. We have reported that LLC-CR cells exhibit higher basal IDO1 activity compared to LLC cells, which are sensitive to the cisplatin [7]. Here, mice were treated with IDOi at 200 mg/kg P.O. once a day for 15 days or with methylcellulose (control group (CTRL)). At harvest, tumor tissues were collected and assayed for IDO1/TDO2 and IDO2 protein expression. IDO1 expression was elevated in mouse allografts with LLC-CR, along with very low to non-detectable TDO2 expression in the control group, as anticipated (Fig. 3A). However, in LLC-CR mice treated with the selective IDO1 inhibitor, we observed a significant increase in TDO2 expression (Fig. 3A; right panel) and changes in IDO2 expression during IDO1 inhibitions (Supplemental Figure S3A). We then created CRISPR-edited* Ido1* knockout in LLC-CR (LLC-CR^SG^) cells to further test IDO1 suppression, and we selected the LLC-CR^SG2^ clone due to the complete knockout efficiency for IDO1 (Supplemental Figure S3B). Again, TDO2 expression was significantly enhanced but did not observe significant changes in IDO2 expression in IDO1 knockout conditions (Fig. 3B). To further identify a potential compensatory mechanism in CR cells and determine if TDO2 inhibition may lead to an increase in IDO1 expression in vivo, we administered a selective TDO2 inhibitor (LM10) instead of IDOi under the same conditions (200 mg/kg P.O. once a day for 15 days). Blocking TDO2 led to an increase in IDO1 expression and activity (higher KYN production) (Supplementary Figure S3C, D), with an increase in IDO2 expression in LLC-CR (Supplementary Figure S3C).

Nevertheless, the question remained whether compensatory increases in TDO2 or IDO2 have functional consequences via higher KYN production. We then assayed for extracellular KYN levels in human cells with IDO1 knockdown (shIDO) and IDO1 mouse-knockout (LLC-CR^SG2^) cells where TDO2 was inhibited (LM10). Extracellular KYN concentrations were significantly suppressed to levels lower than in CS cells with significantly increased TRP concentrations, suggesting that IDO2 may not play an important role in affecting KYN secretion in our CR cell models (Fig. 3C (pink) and Supplemental Figure S4A). To further investigate the observed interplay role of IDO1 and TDO2 enzymes in our CR models, we first examined IDO1 and TDO2 enzyme inhibition in cell culture using our new-in-class IDO1/TDO2 dual inhibitor AT-0174 (see Supplementary Table S1 for chemical formula). In an enzyme inhibition colorimetry assay gauging KYN activity, murine LLC cells transfected with human-IDO1 (LLC-hIDO1) revealed an inhibitory potency (IC_50_) at IDO1 of 0.17 µM. The IC_50_ of the TDO2 enzyme inhibition in murine glioma cells (GL261) transfected with human-TDO2 (GL261-hTDO2) was 0.25 µM (Fig. 3D). Growth inhibitory assay further revealed that both human and mouse-CR cells were more sensitive (~ twofold) to AT-0174 when compared to IDOi (Supplementary Figure S4B). We then assayed for intracellular KYN levels. Cellular KYN was decreased with IDOi, but more significantly upon AT-0174 inhibition (Supplementary Figure S4C) in CR cells. Interestingly, even though CS cell lines do not possess high IDO1 activity; however, we observed a small decrease in intracellular KYN accumulations further indicating that both IDOi and AT-0174 are on-target drugs. Blocking IDO1/TDO2 inhibited the colony-forming ability only in CR cell models, further demonstrating altered metabolism via the dependence of CR cancer cells on these enzymes for survival (Fig. 3E and Supplementary Figure S4D). Collectively, our data support a mutualistic role of IDO1/TDO2 function in CR tumors, and a strong equipotency of AT-0174 for both enzymes.

### Decreased tumor burden upon IDO1 or dual IDO1/TDO2 inhibition in syngeneic and humanized murine models of platinum-resistant NSCLC

**Fig. 4:**
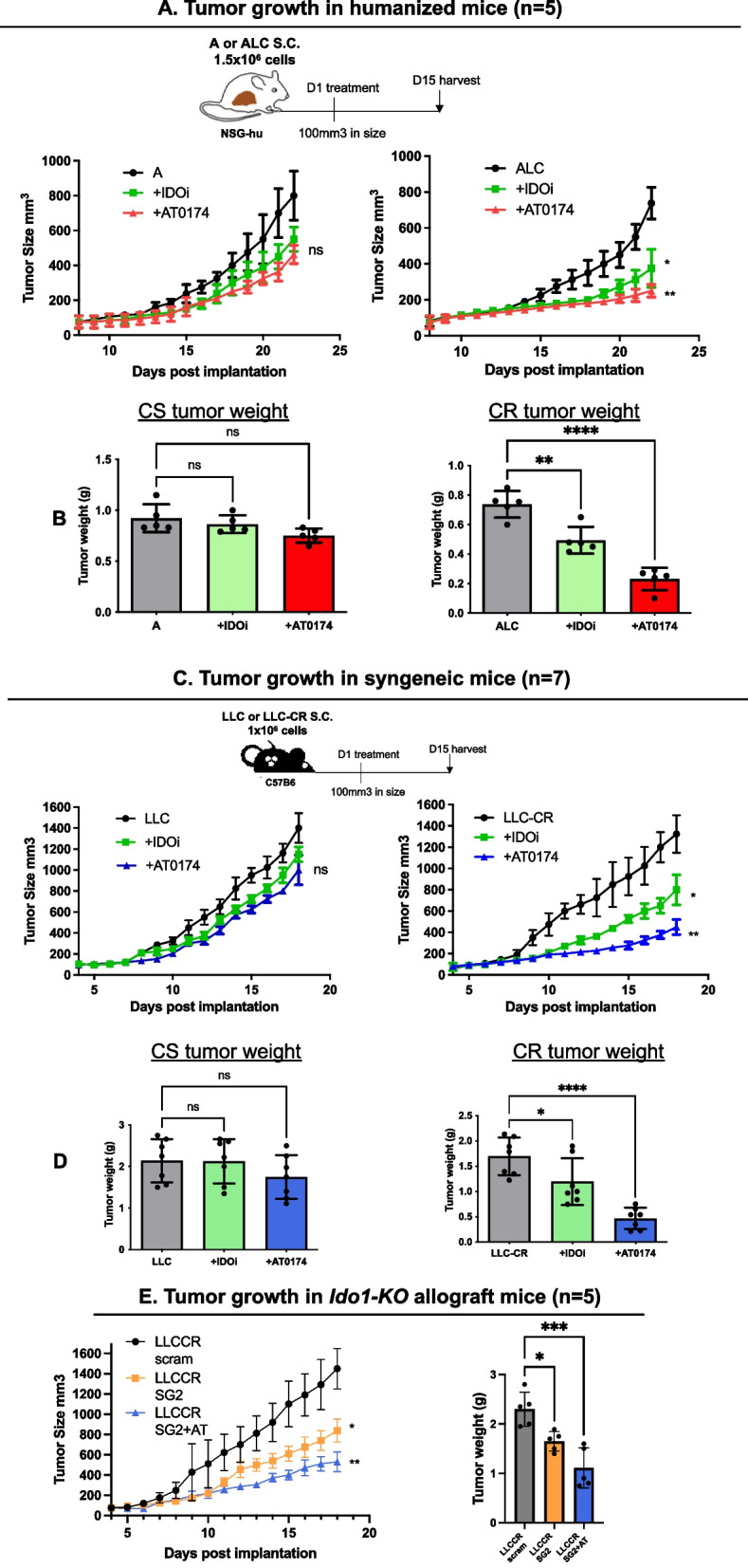
Inhibition of IDO1 and TDO2 suppressed tumor growth in the humanized and syngeneic mouse models. **A **Anti-tumor activity of IDO1 inhibitor or AT-0174 in sensitive vs. resistant tumors using humanized mouse models. **B **IDOi significantly reduced tumor growth in the resistant tumor group (ALC); however, AT-0174 treatment resulted in greater suppression in tumor growth and weight. **C**-**D** Anti-tumor activity of IDOi or AT-0174 in sensitive vs. resistant tumors using syngeneic mouse models. **D** Consistent with the humanized mouse model above, a significant reduction in tumor weight and size was observed in syngeneic mice with resistant tumors treated with AT-0174. **E** Anti-tumor activity of AT-0174 in CRISPR direct *Ido1* KO in LLC-CR tumor (LLC-CRSG2). Tumor burden was significantly suppressed by Ido1-KO and further reduced by AT-0174. Data were analyzed using one-way ANOVA followed by Tukey’s multiple comparisons for tumor growth and Dunnett’s multiple comparisons for tumor weight with *P < 0.05, **P < 0.005, ***P < 0.0005, ****P < *0.0001*

To determine whether our in vitro findings on cancer cell growth and survival can be translated in vivo, we employed humanized mice (NSG-hu CD34^+^), modeling a human immune system, to determine whether blocking the KYN pathway can suppress CR tumor growth. Human CS cell line “A” or CR cell line “ALC” were subcutaneously implanted into the right flank of humanized NSG-hu CD34^+^ mice and orally treated with IDOi (200 mg/kg, 1/day), or with AT-0174 (170 mg/kg, 1/day) for 15 days. IDOi suppressed tumor growth in ALC allografts (Fig. 4A—green line; right panel) with a significant reduction in total tumor weight (Fig. 4B; right panel). However, when comparing IDO1 inhibition to dual inhibition (red line), it was evidenced that dual inhibition was more potent than selective IDO1 inhibition alone in reducing tumor growth and tumor weight of CR tumors (Fig. 4A, B—red).

As in cell lines, an increase in TDO2 expression was observed in LLC-CR inoculated mice treated with either IDOi or AT-0174 (Fig. 3A; right panel). To further examine the mechanistic role of IDO1 and TDO2 in CR tumors in a syngeneic mouse model. Mice-bearing LLC or LLC-CR tumors were treated with IDOi or AT-0174 using the same regimen as in the humanized model described above. Again, selective IDO1 inhibition suppressed tumor growth in CR allografts (Fig. 4C—green line; right panel), with a significant reduction in total tumor weight (Fig. 4D; right panel) when compared with LLC mouse tumors. However, blocking both IDO1 and TDO2 (AT-0174) induced a greater reduction in tumor size (Fig. 4C—blue), and tumor weight in LLC-CR allograft mice (Fig. 4D). Moreover, tumor formation of CRISPR *Ido1*-KO mice (LLC-CR^SG2^) was significantly slower than control LLC-CR, and treatment with AT-0174 further suppressed LLC-CR^SG2^ tumor growth and weight (Fig. 4E). Hence, tumor growth relied on IDO1/TDO2 activity in vivo. Together, our data strongly support the presence of a compensatory mechanism wherein cisplatin-resistant NSCLC tumors can employ IDO1 and/or TDO2 activation to overcome single enzyme pharmacological blockade as therapy.

### Assessment of tumor immune cell profiles and KYN/TRP levels after IDO1 or dual IDO1/TDO2 inhibition in syngeneic and humanized mouse models of platinum-resistant NSCLC

**Fig. 5:**
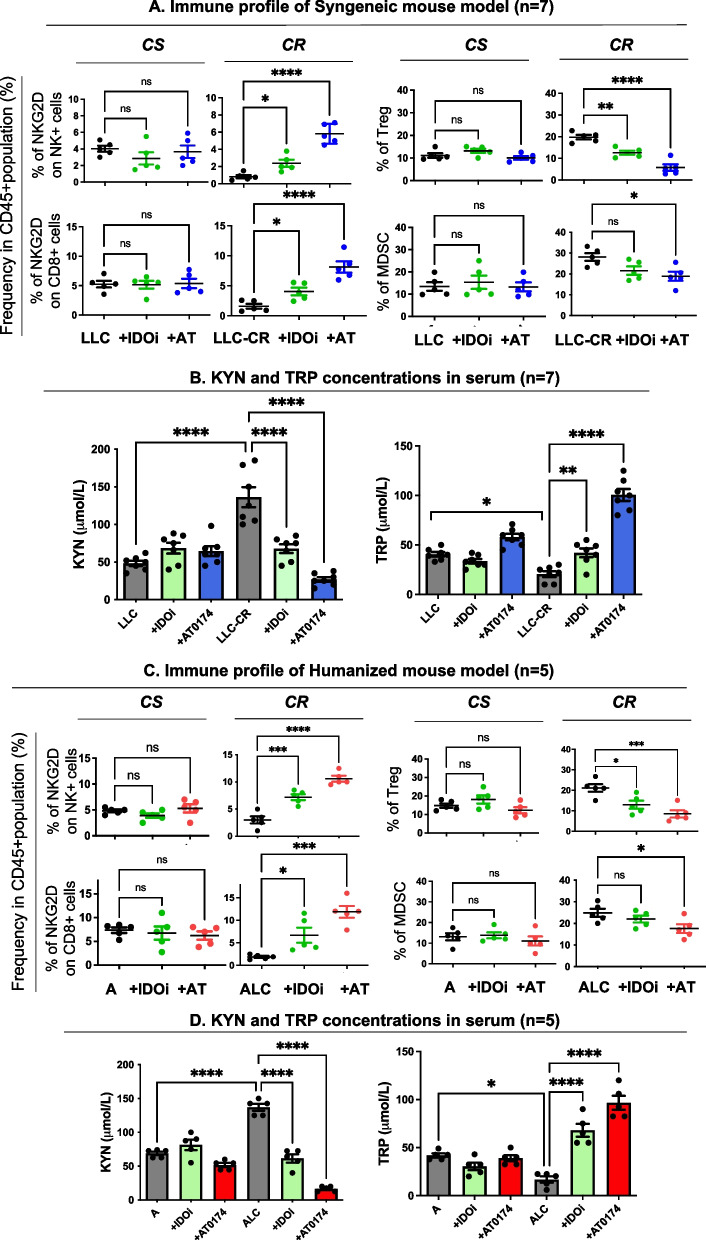
Tumor-infiltrating lymphocytes (TIL) and KYN and TRP level assessments syngeneic and humanized mouse models. Dead cells were excluded with fixable viability dye (FVD). Lymphocytes gated based on SSC-A versus FSC-A and CD45 + /FVD negative were selected (see gating strategy in Supplemental Figure S1B). **A**, **C** Both IDOi and AT-0174 (AT) suppressed T-reg and increased NKG2D frequencies on NKs as well as CD8 + T cells in CR tumors. However, higher NKG2D frequencies on NK and CD8 + T but lower Treg populations were found in the AT-0174 treatment group vs. IDOi. **B**,** D** Higher basal KYN levels of KYN but lower levels of TRP were detected in the serum of CR tumor-bearing mice. Importantly, AT-0174 significantly suppressed KYN secretion and increased extracellular TRP. Data were analyzed using one-way ANOVA followed by Tukey’s multiple comparisons for KYN and TRP concentrations and Dunnett’s multiple comparisons for the immune profile with *P < 0.05, **P < 0.005, ***P < 0.0005, ****P < *0.0001*

Since anti-tumor immunity is reliant on checks and balances between immune effector cells and immunosuppressive cells, we wanted to determine whether inhibiting the KYN pathway can restore anti-tumor immunity in CR tumor models by analyzing tumor-infiltrating lymphocytes (TIL) using flow cytometry (Supplemental Figure S1B for gating strategy in mice). Compared to mice-bearing CS tumors, TIL-Treg and tumor-infiltrating MDSC populations were increased in CR tumors in both syngeneic (LLC-CR) and humanized (ALC) models further confirming the immunosuppressive role of KYN in CR tumors and suggesting that CR tumors may have adapted by evading immune surveillance (Fig. 5A, C; black dots, right panels). Treg populations in CR tumors were significantly reduced by IDO1 inhibition (Fig. 5A, C; green dots, right panel), and more significantly reduced with dual IDO1/TDO2 inhibition in both CR mouse models (Fig. 5A, C; blue and red dots, right panel), leading to increased NKG2D expression on NK and CD8^+^ T populations (Fig. 5A, C; blue and red dots, left panels). It is also important to recognize that IDO1 inhibition, but not dual inhibition, in CS tumors (LLC and A) resulted in a slight increase in Treg and MDSC populations (Fig. 5A, C, green dots, right panel). These data were consistent with the small TDO2 protein expressions shown in LLC (Fig. 3A).

Our data further confirm that KYN not only plays a critical role in the reprogramming of naïve T cells to Tregs but also in impairing NK and T-effector cells’ ability to mount anti-tumor immune responses by modulating the NKG2D receptor in vivo. Serum KYN concentrations were significantly decreased in CR mice after IDO1 inhibition (Fig. 5B, D green bars, left panel), and decreased more after dual inhibition (Fig. 5B, D blue and red bars, left panel). Concomitantly, higher serum TRP levels were found in mice treated with a dual inhibitor when compared to the IDO1 inhibitor alone, confirming a sustained inhibition of TRP catabolism by TDO2 in vivo (Fig. 5B, D; blue and red bars, right panel).

### Effects of IDO1/TDO2 inhibition in combination with PD1 blockage on CR tumor growth and immune cell profiles

**Fig. 6:**
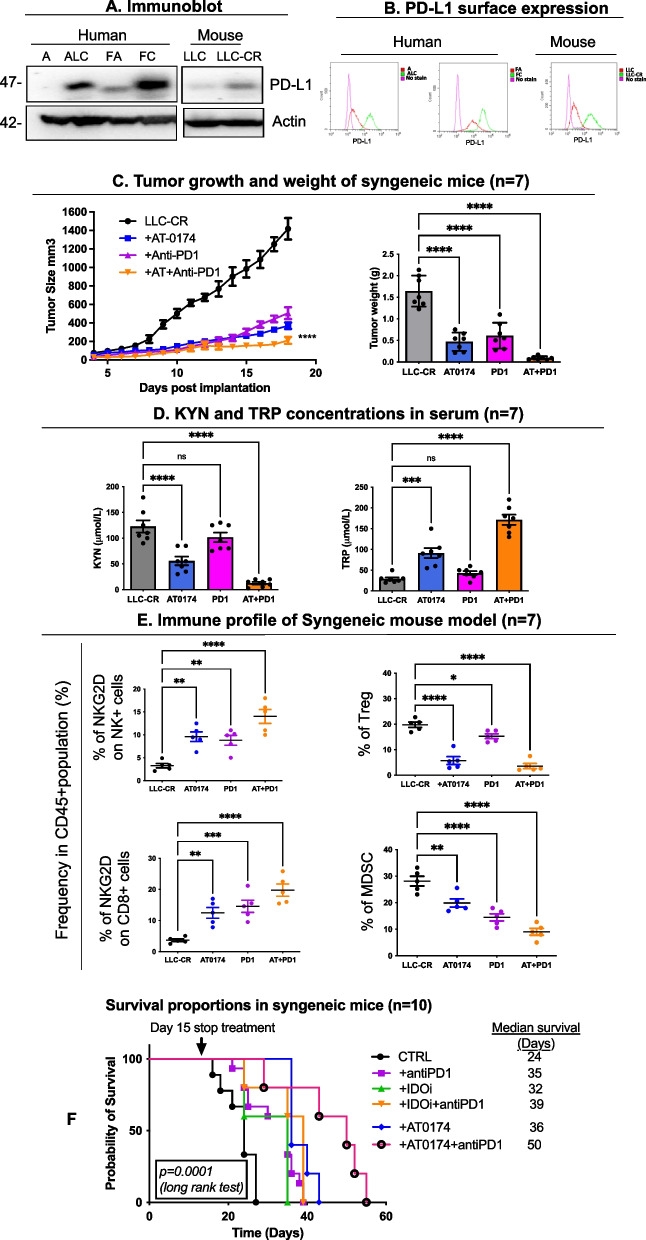
Antitumor efficacy of PD-1 blockage is further enhanced by IDO1/TDO2 inhibition. **A** Immunoblot of PD-L1 in CS vs. CR cells in human and mouse cell lines. **B** Flow cytometry of surface PD-L1 in CS vs. CR cells. CR cells exhibited significantly higher PD-L1 expression. **C** Antitumor efficacy of AT-0174 plus anti-PD-1 antibody in syngeneic mice bearing LLC-CR. Tumor size and weight were significantly decreased upon dual inhibition plus anti-PD-1 treatment. **D** KYN concentrations were significantly reduced and TRP concentrations were significantly higher in mice treated with AT-0174 compared to the control. This result was maintained and further heightened by combining AT-0174 and anti-PD-1 antibody. **E** Flow cytometry characterization of immune profile in tumors. Decreased T-reg populations were found in both single and combination treatments in syngeneic mice bearing LLC-CR. AT-0174 enhanced NKG2D frequencies on NK and CD8 + T cell populations. Significant decreases in MDSC populations were found in combination treatments. **F** Kaplan–Meier survival curves for the in vivo assessment of AT-0174 alone, IDOi alone, and combination treatment with anti-PD-1 antibodies were assessed in the syngeneic mouse model. Since tumor size was significantly reduced, treatments were stopped on day 15, and efficacy was determined by comparing the median times to the endpoint (either death or euthanasia for advanced tumor progression). Data were analyzed using one-way ANOVA followed by Tukey’s multiple comparisons for tumor growth and Dunnett’s multiple comparisons for the tumor weight, immune profile, and KYN/TRP concentrations with *P < 0.05, **P < 0.005, ***P < 0.0005, ****P < *0.0001*

Previous studies have reported higher expression of PD-L1 (program death receptor ligand-1) in many solid tumors, including NSCLC, upon treatment with platinum chemotherapeutic agents [25, 26, 33]. To ascertain that PD-L1 protein expression is increased in CR compared to CS cells, we analyzed baseline PD-L1 protein expression in our model. PD-L1 expression (Fig. 6A, B) was increased in all of our CR cell models (human and mouse), thus providing a rationale for combining the use of dual IDO1/TDO2 inhibition with PD1 blockade (anti-PD1 antibody), which we subsequently examined in the LLC-CR syngeneic mouse model. Intraperitoneal injection of anti-PD1 antibody (10 mg/kg, every 3 days) in combination with AT-0174 resulted in greater suppression of CR tumor growth than PD1 blockage alone, or AT-0174 treatment alone (Fig. 6C). Significantly lower tumor weights were found in all of the treatment groups, with IDO1/TDO2 inhibition plus PD1 blockade yielding the greatest reductions (Fig. 6C; right panel). This treatment combination did not affect tumor growth and weight in a sensitive LLC mouse model, demonstrating the applicability of dual inhibition + anti-PD1 blockade in CR tumors and not sensitive models (Supplemental Figure S5).

AT-0174 treatment in CR tumor-bearing mice resulted in significantly decreased KYN in serum and significantly elevated TRP (Fig. 6D; orange). Correlating with these changes, combination treatment also significantly increased NKG2D frequency on NK^+^ cells and CD8^+^ T cells. Immunosuppressive cell populations (Tregs and MDSCs) peaked in high KYN and low TRP serum conditions, and decreased in all treatment groups, compared to no treatment, with the most significant decrease seen with AT-0174 + PD1 blockade (Fig. 6E; orange). Next, we analyzed the survival benefit of combination treatment on syngeneic mice bearing CR tumors. Comparing dual IDO1/TDO2 inhibition (AT-0174) with IDO1 inhibition when each was given alone, we observed that dual inhibition increased median survival (36 days) more than selective IDOi (32 days) (Fig. 6F). As depicted in Fig. 6F, the median survival of mice treated with AT-0174 or anti-PD1 was 36 and 35 days respectively, increasing to 50 days upon AT-0174 + PD1 antibody treatment. Collectively, our results show that the AT-0174 increases the antitumor effect of PD1 blockade on cisplatin-resistant, PD-L1-expressing, NSCLC in vivo. These data suggest platinum-resistant tumors have an IDO1- and TDO2-dependent metabolic impact (higher KYN/lower TRP) on the tumor microenvironment by promoting immunosuppression (higher Treg and MDSC frequencies, lower % of NKG2D NK^+^ and CD8^+^ cells).

## Discussion

As platinum-resistant NSCLC tumors progress, they exhibit a metabolic shift from glycolysis to oxidative metabolism for survival and slower growth, eventually moving toward a reliance on amino acids. We have shown that an increase in TRP catabolism resulted in an upregulation of KYN production, supporting an immunosuppressive microenvironment in CR tumors [7] (Fig. 1H). Importantly, we have reported that the KYN/TRP ratio, indicative of IDO1 activity, is elevated in serum from patients who failed platinum treatment [7]. Although our previous clinical sampling was small, it was important to note that our cell culture models correlate well to clinical findings of KYN/TRP ratios among NSCLC patients, thus validating the use of our models for these current experiments. Based on these findings, we expanded our studies toward the assessment of immunosuppression and evaluated tumor-infiltrating lymphocyte (TIL) populations and tumor growth in vivo. Our treatment schemes were designed to improve inhibition of the KYN pathway in tumors by avoiding the reported clinical failure to enhance anticancer activity with selective IDO1 inhibitors (e.g., epacadostat) when used in combination with pembrolizumab [32].

**Fig. 7:**
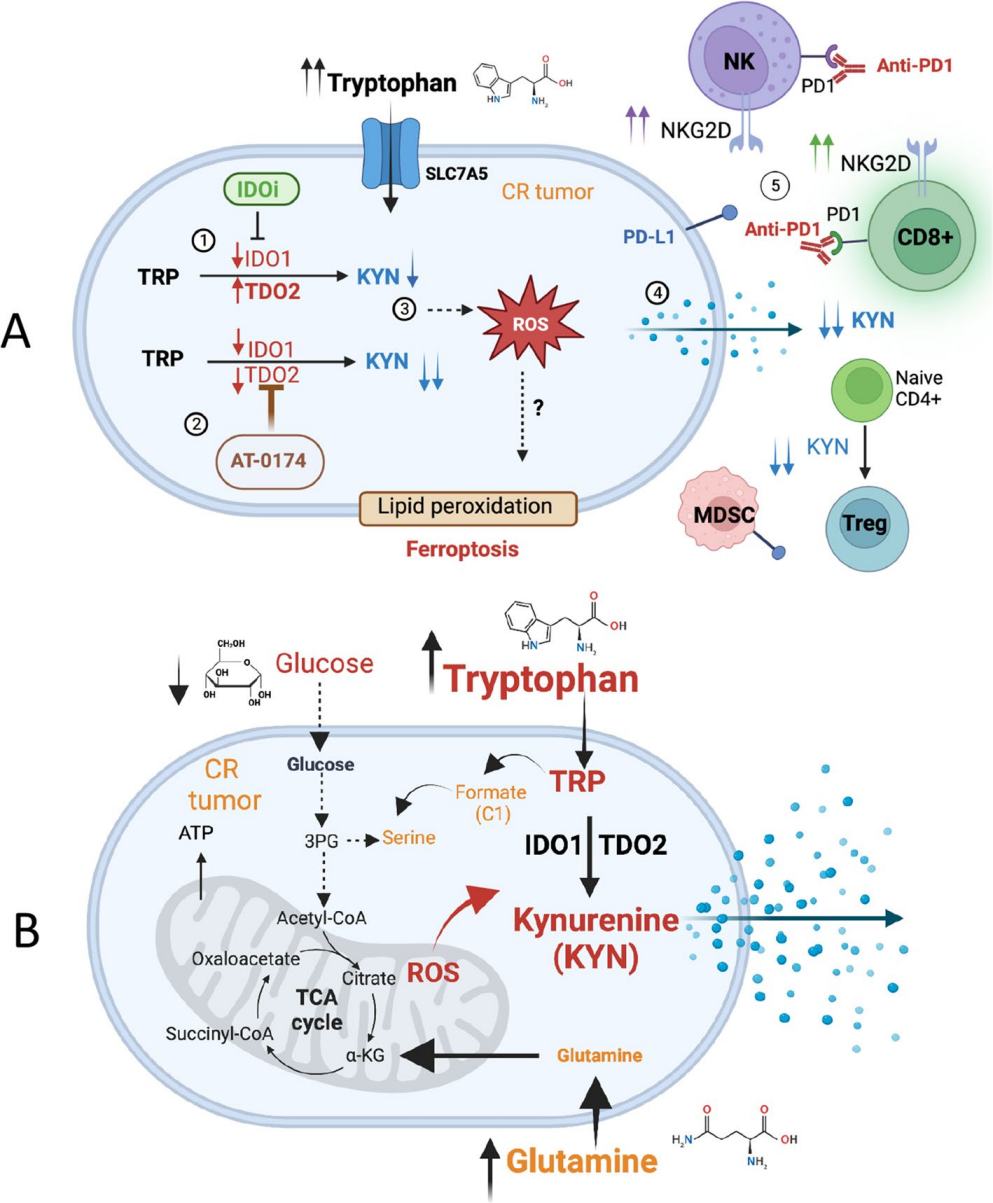
**A** Dual role of IDO1/TDO2 in KYN-mediated cisplatin-resistance. *(1)* Inhibition of IDO1 results in compensatory TDO2 expression for that loss of function. *(2)* Dual IDO1/TDO2 inhibition (AT-0174) is crucial for the inhibition of KYN in CR cells. *(3)* Reduced KYN (a potent inhibitor of ferroptosis?) leads to increase ROS and lipid peroxidation. *(4)* Suppressing KYN production reverses the immunosuppressive environment by enhancing NKG2D on NK and CD8 + T cells and suppressing Tregs. *(5)* AT-0174 + PD-1 blockade further enhances anti-tumor immunity in platinum-resistant NSCLC. **B** CR cells undergo metabolic reprogramming wherein tumors become more reliant on oxidative metabolism (OXMET). TRP can be substituted for serine as a one-carbon donor [49]. We postulate that the depletion of glycolytic-derived serine will hypersensitize CR tumors to IDO1/TDO2 inhibition

Lung tumors predominately express IDO1 but also low levels of another heme-containing enzyme that catabolizes TRP, namely TDO2 [34]. TDO2 expression in CR cells was very low to non-detectable when compared to IDO1 expression, leading to the idea that IDO1 may be more significant for metabolism in these tumor types. However, we have shown here that inhibition of IDO1 resulted in TDO2 compensating for that loss of function. In support of a compensatory functional role for TDO2 in NSCLC, KYN secretion was found in IDO1 knockout mice leading to lung metastasis [35]. This finding was striking, and we found that treatment with a TDO2 inhibitor alone can augment IDO1 expression and activity in both CS and CR tumors (Supplemental Figure S3C, D). Thus, given the induction of TDO2 upon IDO1 inhibition or vice versa, it can be argued that dual IDO1/TDO2 inhibition may be necessary for the inhibition of KYN and reversing immunosuppressive tryptophan catabolism (Fig. 7A). We also noted that different results of TDO2 expressions were obtained between chemical (epacadostat) vs. genetic (shIDO1/CRISPR*-Ido1*) inhibitions of IDO1. As shown in Fig. 2A, CR cells were treated in vitro with epacadostat for 72 h and assayed for TDO2 protein expression. However, for the shIDO1 experiment, clones were selected after many days from a stable knocked down. The in vivo data depicted in Fig. 3A further support this explanation by showing high TDO2 expression in LLC-CR tumors after 15 days of epacadostat pharmacological treatment. These in vivo data, along with the in vitro data suggest that TDO2 expression is not sudden or perhaps less responsive than IDO1, but needs time to propagate in NSCLC. These findings warrant further investigation since such concepts are supported by other studies wherein immediate molecular or growth inhibitory effects are not observed, and varying dosage/timing is needed to elicit the effect.

Dual inhibition of TRP catabolizing enzymes has been previously suggested [36, 37] and the development of pan-inhibitors blocking IDO1/2 and TDO2 has been reported [38]. However, targeting IDO2 remains controversial. Deletion of IDO2 has reduced the tumor volume in a mouse model of the Lewis lung carcinoma and in *Kras*^*mut*^ pancreatic ductal adenocarcinoma [39, 40]. However, IDO2 activity has induced an inflammatory response in an arthritis mouse model [11] and was protective in a psoriasis mouse model [41]. In humans, the absence of IDO2 increases the risk of developing NSCLC [42]; however, in another cancer study, lower expression of IDO2 was correlated with good prognosis [43]. In our CR model, we did not observe a significant change in IDO2 expression before and after dual inhibitors treatment. At present, we still do not understand the exact impact and important consequences of IDO2 in the TME. We however recognize that TRP catabolic activity by IDO2 is very poor [29] in our CR model, and may represent a pseudoenzyme lacking relevant catalytic activity in the KYN pathway [42].

We demonstrated that increased KYN concentrations suppress NKG2D expression in vitro and in vivo CR tumor models. This is important since others have reported that KYN inhibits the surface expression of NKG2D-activating receptors and regulates NK cell function [21]. Thus, NK cells may be an additional target for IDO-mediated suppression in CR tumors. Along with our finding that platinum-resistant NSCLC tumor cells express more PD-L1 than platinum-sensitive cells, we postulated that PD-1 blocking should have a pronounced effect on inhibiting CR tumor growth. Indeed, others have shown that anti-PD-1 antibodies can also increase the NKG2D expression [44]. Here, we demonstrated that the combination of a dual inhibitor, AT-0174, plus PD-1 blockade markedly enhanced NKG2D frequency on NK and CD8^+^ T cells which led to a reduction in tumor growth and tumor weight compared to monotherapy, and prolonged survival. Regarding the potential role of IDO1 activity affecting MDSCs, we found that CR tumors with greater IDO1 activity, possessed higher tumor-infiltrating MDSC populations when compared to CS tumors. This finding was consistent (CS vs. CR) in our syngeneic and humanized mouse models. Interestingly, another study has shown that intratumorally, IDO1 induced immunosuppression by expanding, recruiting, and activating MDSCs and leading to immunotherapy resistance [15]. This finding adds another mechanistic role for IDO1-mediated immunosuppression not specifically reliant on Tregs alone.

 The combination of AT-0174 plus anti-PD-1 treatment approximately doubled survival over the control condition in mice harboring cisplatin-resistant tumors, a finding that further substantiated the reliance of these tumors on altered metabolism (KYN) for survival. This degree of effect was especially notable, given that co-treatment occurred for only 15 days and then ceased and was not continued throughout the duration of the study in this instance. Continuous co-treatment would be expected to confer an even greater survival benefit, and such studies are currently underway. This finding of synergy is novel among platinum-resistant NSCLC and aligns with extended therapeutic effects reported in various clinical findings of immuno-therapies including immune checkpoint inhibitors. A major concern in the application of immunotherapy combinations is the potentiation for excessive immune activation (higher CD8^+^ and NK cells with lower Tregs and MDSCs) leading to autoimmune effects [45]. For this reason, we observed the animals treated herein carefully, and we did not note any deleterious behavioral or morphological sequelae during the course of these experiments. Although it is premature to speculate on the safety profile of the experimental combinations presented in this study, the dual inhibitor AT-0174 was well tolerated and appears to be non-toxic to normal tissues at efficacious doses when administered to *Cynomolgus* monkeys (data communicated by Antido Therapeutics, based on formal preclinical analyses of drug candidate safety in primates).

There is supporting evidence for the presence of an immune-depressed/inhibited effector T cell population among patients receiving platinum-based therapies [46, 47]. Our work supports the notion that the efficacy of anti-PD-1 therapy is limited by an inability to adequately activate immune effector cell populations to mount anti-tumor immunity due to the effects of metabolic alterations in platinum-resistant NSCLC. The results of the metabolic alterations that we have characterized, such as a high KYN/TRP ratio, have been previously shown to be significantly associated with tumor progression and resistance to anti-PD1 therapy [48]. We have previously reported that NSCLC patients possessed higher KYN levels as compared to healthy control subjects [7] and showed here that CS tumors also possess high KYN. However, KYN concentrations increased greatly in patients who failed platinum therapy compared to sensitive patients, or in CR tumor-bearing mice. But at this time, there is inadequate data available to identify a KYN/TRP ratio value in serum that can be used as a diagnostic biomarker for resistance to therapy. We propose the need for additional studies of serum KYN and TRP levels among treatment naïve and previously treated populations to derive an informative value. This could result in the use of KYN and TRP serum levels as surveillance markers during the course of the standard-of-care chemo-radiation, and immunotherapies to identify a patient population with tumors reliant on the KYN pathway for growth and survival.

Our new findings support a new paradigm of platinum-resistant lung tumors that utilize both IDO1/TDO2 to survive and escape immune surveillance as a consequence of KYN metabolites. Our studies further support the potential therapeutic efficacy of dual IDO1/TDO2 inhibition as a part of immuno-therapeutic treatment that enhances tumor immune surveillance and blocks tumor metabolism-related survival mechanisms in platinum-resistant NSCLC tumors.

## Study limitations

Over the past decade, we have shown that NSCLCs acquiring resistance to cisplatin undergo metabolic alterations that increase tumor reliance on oxidative metabolism (OXMET) instead of glycolysis. Switching to OXMET CR cells increases cellular metabolic demand and outstrips glutamine supply, making glutamine conditionally essential for tumor survival. Besides glutamine, CR cells are also auxotrophic for arginine and sensitive (viability) to blockage of the kynurenine pathway. Our study is limited by the question of why TRP metabolism plays an important role in CR cell survival. Recent studies showed that IDO1-dependent tryptophan metabolism is a bona fide one-carbon source for folate-dependent nucleotide synthesis [49] because TRP can be substituted for serine as a one-carbon donor, with serine deprivation improving the anti-tumor activity of IDOi. Since CR cells rely on OXMET rather than glycolysis, serine production (from glycolysis) should be limited, thus making CR cells hyper-sensitive to dual inhibitors (Fig. 7B). The Newman et.al. discoveries, as well as ours, may explain why CR cells require one tryptophan oxidation system “on” at all times for growth and proliferation. Our findings at this point are not exhaustive with respect to answering these two questions.

Another limitation is that dual inhibitors can selectively target CR cells, but we have not evaluated the mode of cell death and how KYN is being transported during this process. A recent study by Fiore et al. showed that KYN and its immediate downstream metabolites are potent inhibitors of the ferroptosis/redox stress [9]. Hence, suppressing KYN by IDO1/TDO2 mediated inhibition should potentially promote ferroptosis or more redox stress. Importantly, Fiore et. al. showed that KYN is imported into cells by SLC7A11 (xCT/cystine/glutamate antiporter). These findings are intriguing and aligned with our model since we have previously reported that CR cells are hypersensitive to xCT antiporter inhibition [5]. Thus, blocking SLC7A11 should result in significant ferroptosis cell death in CR tumors. We have not collected data on these mechanisms of cell death at this time. In addition, we have also reported that CR cells express higher levels of phospho-AhR (aryl hydrocarbon receptor) protein compared to cisplatin-sensitive cells in the nucleus [7]. This condition can potentially have various transcriptional impacts that we can investigate further. Also, KYN activated AhR which led to the induction of the tryptophan transporter SLC7A5, creating a positive feedback loop [7, 50]. Recently developed AHR antagonist has shown efficacy in melanoma [51] and could potentially have an impact on the platinum-resistant NSCLC. These highlighted findings provide additional roles for KYN, which are not limited to reprogramming T-reg in the TME.

## Material and methods

### Test compounds

The dual IDO1/TDO2 inhibitor AT-0174, a novel, orally-bioavailable, isoxazolopyridin-3-amine compound, was provided for use by Antido Therapeutics (Melbourne, Australia). AT-0174, a proprietary, novel, small molecule was developed as part of a novel compound chemical synthesis and screening program, from an isoxazolopyridine-based pharmacophore (Patent# US20210145839A1) by Antido Therapeutics (Supplementary Table S1). The compound was stored at − 20 °C and formulated as a solution in 95% water + 5% methylcellulose (0.5%) to provide a fine suspension suitable for oral gavage treatment. The selective IDO1 inhibitor (epacadostat, cat no. T3548) or TDO2 inhibitor (LM10, cat no. T4410) were obtained from Targetmol and stored at − 20 °C. Epacadostat and LM10 were also formulated in 95% water + 5% methylcellulose (0.5%) for in vivo oral administration to mice. Antibody against PD-1 (anti-PD-1 clone RMP1-14, cat no. BE0146) was obtained from BioXCell and stored at 4 ˚C. Antibody was administered intraperitoneally every 3 days. Normal saline was used as a control injection.

### Cell lines and reagents

A pair of CS vs. CR human NSCLC cells (A vs. ALC) and a pair of CS vs. CR mouse Lewis lung cells (LLC vs. LLC-CR) were used. Cell line “A” was established from the pleural fluid of a patient with adenocarcinoma. Cellular characteristics have been previously characterized [52–55] and routinely tested for mycoplasma infection (MycoAlert, cat no LT07-318). Mouse cisplatin-resistant cell line (LLC-CR) was derived from LLC (mouse Lewis lung cancer cells; ATCC, Manassas, VA, USA). CR cells were generated by treatment with increasing doses of cisplatin intermittently. LLC-CR clones were maintained in half GI_50_ dosage concentration. Briefly, cells were seeded (4 × 10^4^) in 6-well plates and treated with clinical-grade cisplatin (Miami VA Hospital) for 24 h with the 50% growth inhibition (GI_50_) concentration of 0.4 µg/ml. The cultures were observed daily and allowed to grow until they reached an initial cell density. One to twofold increase in GI_50_ concentration was observed within 1–2 weeks. After the cells recover from cisplatin toxicity, they were treated again with an increasing dosage of cisplatin for 24 h. These cells are seeded at 500–1000 cells per dish and cultured for 5–7 days. Clones are selected and expanded as well as tested for cisplatin sensitivity. Similar processes of exposure are carried out for a third and fourth time to generate higher resistant clones. In the end, the resistance clones were maintained in media completed with 1 µg/ml (half of the GI_50_ dosage) of cisplatin. CR cells were developed to possess 3–fivefold resistance to cisplatin and carboplatin (~ 2.5 µg/ml and ~ 1.5 µg/ml, respectively), with concomitantly increased IDO1 activity and sensitivity to the IDO1 inhibitor [7].

### Animal studies

Procedures and mice protocol were approved by the Institutional Animal Care and Use Committee (IACUC) of the Miami VA Healthcare System (Animal Welfare Assurance Number: A3739–01). C57BL/6 was raised in the Miami VA animal facility LLC or LLC-CR (1 × 10^6^) cells were subcutaneously injected into the flank of C57BL/6 male and female mice (age 6–8 weeks). Human cell lines A or ALC (1.5 × 10^6^) were subcutaneously injected into the flank of NOD-*scid* IL2Rgamma^null^ mice also known as NSG-hu CD34^+^ humanized (Hu-NSG) mice (5 mice per group, age 25 weeks; Jackson Laboratory, Bar Harbor, ME; USA). The grafted tumor was allowed to grow to an average tumor size of 150 mm^3^ before being treated with IDOi or with LM10 at 200 mg/kg daily, or with AT-0174 at 170 mg/kg daily. Compounds were prepared every 5 days and were administered via oral gavage (P.O.). Methylcellulose (95 + 5%) was used as a control treatment. Tumor growth was evaluated every 2 days by measuring tumor volume with calipers according to the following formula: tumor volume = width^2^ × length × 0.5. At the end of the study, mice were sacrificed and the blood and tumor were collected and processed for KYN and TRP measurement and immune cell assessment respectively.

#### Humanized mice

NSG mice carry two mutations on the NOD/ShiLtJ genetic background which include a severe combined immune deficiency (SCID) and a complete null allele of the IL2 receptor gamma chain (IL2rg^null^). The SCID mutation is in the DNA repair protein *Prkdc* and causes the mice to become B and T cell-deficient. The IL2rg^null^ mutation prevents cytokine signaling and leads to a deficiency in functional mice’s NK cells. NSG mice were engrafted with human CD34 + hematopoietic stem cells. Approximately, 1 × 10^5^ of freshly isolated CD34 + HSCs, over 90% pure, were injected intravenously into mice 24 h after irradiation. The engraftment levels of human CD45 + cells and human immune cell populations, including CD45 + , CD3 + , and CD4 + CD8 + T cells, B cells, NK cells, MDSCs, and other lineage-negative cells were determined in the peripheral blood, bone marrow, and spleen tissue. Mice that had over 25% human CD45 + cells in the peripheral blood were considered humanized. All Hu-NSG mice were confirmed for humanization before tumor xenograft by Jackson Laboratory.

### Transwell co-culture experiments

Human peripheral blood mononuclear cells (hPBMCs) were activated for 48 h before co-culture using anti-CD2/28 monoclonal antibodies with IL-2 as mentioned above. On day 3, cells were washed and incubated with 1 µm of carboxyfluorescein succinimidyl ester (CFSE) (BioLegend, cat no. 423801) at 37 °C for 7 min then thoroughly washed with staining buffer before in vitro co-culture. The proliferation rate of lymphocytes was determined by assessing the reduction of the intensity of the fluorescent cell-permeable dye CFSE. For co-culture experiments, 24-well transwell chambers with a 0.4-µm porous membrane (Corning-Costar) were used. Briefly, CS or CR cells (5 × 10^4^/well) were plated underneath the transwell chamber for 4 h, and then 1 ml of activated-CFSE labeled human peripheral blood mononuclear cells (1 × 10^5^/ml) was added to the inner chamber. After 48 h, hPBMCs were transferred to a V-bottom plate (Greiner), washed twice with ice-cold PBS containing 0.5% bovine serum albumin (BSA) (Sigma), and incubated for 5 min at 4 °C with Fc-receptor blocking reagent (BioLegend, cat no. 422302) and stained for indicated immune cell populations.

### Lymphocyte and MDSC cells expansion

Human PBMCs (5 × 10^7^) were obtained from (Stemcell Technology, cat no. 70025). Cells were re-suspended with complete medium, RPMI1640 medium containing 10% FBS, 100 U/ml penicillin–streptomycin, and 2 mM glutamine, then plated onto the six-well dishes at the concentration of 2 × 10^6^/well. For NK and CD8 T cells activation, hPBMCs were stimulated with anti-CD2 (1 µg/ml)/anti-CD28 (1 µg/ml) monoclonal antibody (BioLegend) with 20 ng/ml of IL-2 (Stemcell Technologies) at the indicated culture period with a concentration. For MDSCs activation, hPBMCs were primed with 10 µg/ml of phytohemagglutinin (PHA) (Sigma) + 20 ng/ml of IL-2. Activated hPBMCs were collected and sorted for NK(CD3-CD56 + NKG2D +) and CD8 + T(CD3 + CD8 + NKG2D+) cell populations using ASTRIOS EQ MoFlow cell sorter (Beckman Coulter).

### Flow analysis

Tumors were collected from mice and homogenized using syringe plungers. Mesh filters (40 µM) were placed on 15-ml or 50-ml tubes and the tissue suspensions were filtered. Cell filtrates were spun down at 1000 g. Red blood cell lysis buffer (1% ammonium oxalate) was added to the cell pellets and resuspended. Cell pellets were washed using PBS and isolated. The following procedures were performed in phosphate-buffered saline (PBS) containing 2% FBS and 1 mM EDTA (staining buffer). Cells were isolated using EasySep™ Mouse TIL (CD45) Positive Selection Kit according to the manufacturer’s protocol (STEMCELL Technologies, cat no. 100–0350). To assess viability, cells were stained with LIVE/DEAD fixable violet dead (FVD violet) cell stain kit (1:1000) according to the manufacturer’s protocol (Life Technologies, cat no. L34955). To detect regulatory T cells and NK cells (NKs), EasySep™ Mouse CD25 Regulatory T Cell Positive Selection Kit (STEMCELL Technologies cat no. 18782) and EasySep™ Mouse CD49b Positive Selection Kit (STEMCELL Technologies, cat no. 18755) were used, respectively. Humanized mouse cells were isolated using EasySep™ Release Human CD45 Positive Selection Kit (STEMCELL Technologies, cat no.100–0107). To detect human regulatory T cells (Tregs), CD4 + CD25 + and CD4 + CD25^neg^ T-lymphocytes were isolated by positive selection, and the CD127^dim^ subset of CD4 + CD25 + T regulatory cells was isolated by a subsequent negative selection using the Human CD4 + CD127^lo^CD25 + Regulatory T Cell Isolation Kit (STEMCELL Technologies, cat no. 18063) according to the manufacturer’s protocol. NK cells (NKs) were isolated by EasySep™ Human CD56 Positive Selection Kit II (STEMCELL technologies, cat no. 17855), respectively. To detect human MDSC, HLA-DR^lo^CD14-CD11b + CD33 + were gated. To detect mouse MDSC, F4/80^lo^Ly6C + CD11b + Gr1 + were gated.

The following procedures were performed in phosphate-buffered saline (PBS) containing 2% FBS and 1 mM EDTA (staining buffer). To label cell-surface molecules, cells were incubated for 30 min at room temperature in the dark with specific antibodies, washed, and fixed with 2% paraformaldehyde. For simultaneous detection of the intracellular molecule, after fixed, cells were permeabilized with True-Nuclear™ Transcription Factor Buffer Set according to the manufacturer’s protocol (BioLegend, cat no. 424401) and then incubated in a water bath at 37 °C for 30 min with anti-FoxP3-Alexa Fluor® 488 MAb. All flow antibodies were diluted at 1:100 dilution and were obtained from BioLegend (see the list of antibodies with RRID in Supplementary Table S2). Fluorescence minus one (FMO) was used to identify gating boundaries. Flow analysis was done using the CytoFLEX Flow Cytometer equipped with 3 lasers (488 nm blue, 405 nm violet, and 638 nm from Beckman).

### Intracellular extracellular KYN and TRP measurements

Biochrom 30 + Series amino acid analyzer (ion-exchange chromatography, Biochrom, Ltd., Cambridge, UK) was used to analyze free amino acids in samples. Cells, tumor tissues, or blood were used for amino acid analysis. Blood was collected via cardiac puncture from mice at the time of sacrifice and serums were extracted. Cell pellets were collected and sonicated in 100 µL of cold 50 mM of MES (2-(N-morpholino) ethanesulphonic), pH 6–7, containing 1 mM EDTA. Briefly, serum extract (200µL) or cells’ lysate (100 µL) with an equal volume of 30% v/v deproteinization agent (sulfosalicylic acid; Sigma, cat no. S2130) were mixed and vortexed for 10 s. Samples were then incubated at 55 °C for 20 min and spun down at 2500 g for 20 min at 4 °C. The supernatant was collected, filtered thru 0.22 micron, and injected into the Biochrom 30 + . Values were reported compared to normal ranges in controls. Samples used in ion-exchange chromatography are separated analytically with an ion-exchange column and buffers of increasing pH and ionic strength. After post-column derivatization with Ninhydrin, and colorimetric intensity recordings at 570 nm and 440 nm, the amino acids separated by chromatography were detected. The amino acid concentration was calculated by comparing the peak area of a particular amino acid to the peak area of an internal standard-AEC of known concentration and then multiplying by its specific response factor from calibration.

### Cellular IDO1 enzyme inhibition assay

Lewis-lung carcinoma cells (LLC) stably transfected with the expression vector F279-V5/hIDO1 (Gateway™, Invitrogen) containing human IDO1 under the control of the CMV promoter, were used for a cell-based assay of IDO1 enzyme activity (LLC-hIDO1). AT-0174 were solubilized in 0.5% (v/v) DMSO and incubated (24 h; 37 ˚C) with 2 × 10^4^ LLC-hIDO1 cells/well in α-MEM medium (containing 10% fetal calf serum, 10% penicillin (100 U/ml), streptomycin (100 μg/ml) and puromycin (2.5 μM) and 49 µM tryptophan). Incubation supernatants (120 μl) were treated for 20 min at 60 ˚C with 10% trichloroacetic acid and spun (2500 g, 10 min) before mixing with p-DMAB (20 mg/mL of para-Dimethylaminobenzaldehyde in glacial acetic acid). After 10 min incubation in the dark, absorbance was read at 490 nm on a SpectraMax M2 (Molecular Devices) to assay kynurenine content. The concentration of kynurenine in the supernatants was determined from a calibration curve of the kynurenine standard, to provide a measure of IDO1 enzyme inhibition in the cells.

### Cellular TDO2 enzyme inhibition assay

The murine glioma cell line GL261 was engineered to overexpress full-length human TDO2 under the control of a strong cytomegalovirus promoter (GL261-hTDO2 cells; Invitrogen). AT‑0174 were solubilized in 0.5% (v/v) DMSO and incubated for 24 h (37 ˚C, 5% CO2) with 1 × 10^4^ GL261-hTDO2 cells/well in α-MEM medium containing 10% fetal calf serum and 400 µM tryptophan. The incubation supernatant was treated for 20 min at 60 ˚C in 40% trichloroacetic acid before treatment with Ehrlich’s Reagent (dark, room temperature, 10 min), and measurement of kynurenine was determined from the absorbance at 500 nm SpectraMax M2 (Molecular Devices), compared against a kynurenine standard curve, to provide a measure of TDO2 activity.

### Clonogenic assay

The human CS or CR cells were treated with 25 μM of AT-0174 or IDOi for 72 h and then resuspended and re-seeded onto six-well plates at a density of 1000 cells/well. After being cultured for 12 days, cells were fixed with 4% paraformaldehyde for 10 min, stained with 1% crystal violet for 20 min, and washed with PBS.

### Knockdown and CRISPR/Cas9-directed genome editing of Ido1

For stable knockdown (shRNA) of IDO1, CR (ALC) cells were transfected with 1 μg of pGFP-C-shLenti plasmid expressing shIDO1 (NM_002164) or non-effective 29-mer scrambled shRNA control (Origene, cat no. TL312147) using lipofectamine 3000 (Thermo Fisher, Waltham, MA, USA) as transfection reagent in Opti-MEM medium. After 24 h, transfection medium Opti-MEM was exchanged to RPMI1640 containing 5 µg/ml of puromycin (Sigma, St. Louis, MO, USA). GFP as a reporter was used to evaluate target gene knockdown efficiency. See Supplemental Table S3 for short hairpin sequences. As for gene knockout, we have generated CRISPR*-Ido1* knockout (*SG1&2*; NM_008324) in LLC-CR cells (LLCCRSG1 and SG2). Using the protocol of the manufacturer, *Ido1* vector clone *1* or *2* in pCas-Guide CRISPR vector was incubated with linear donor DNA containing LoxP-EF1A-tGFP-P2A-Puro-LoxP (OriGene, cat no. KN508091). LLC-CR were transfected with CRISPR plasmids using Lipofectamine 3000 (Invitrogen). Clones were harvested after being selected using 2 μg/ml of puromycin. To evaluate the efficiency of CRISPR-directed *Ido1* knockout in the total targeted population, GFP + cells were sorted by cell sorter. These *SG1 and SG2* clones were tested for viability and clonogenic survival as well as cisplatin sensitivity. All cell lines will be authenticated by short tandem repeat DNA profiling and monitored to be free of mycoplasma. pCas-Guide-CRISPRi-Scramble was used as control. See Supplemental Table S4 for single guide RNA sequences.

### Cytokine assay

To detect multiple cytokines simultaneously, we used the LEGENDplex™ human CD8/NK (13-plex) panel (BioLegend, cat no. 740267). LEGENDplex™ is a bead-based immunoassay that utilizes the same basic principles as sandwich immunoassays. This system simultaneously detected and quantified 13 cytokines in the same sample. Briefly, culture supernatant samples were collected without dilution. Samples were mixed with assay buffer + human (13-plex) mixed bead and plated on the 96-well filter plate. Sealed and shake the plates @500 rpm for 2 h at room temperature. Washed the plate by adding 200 µL of 1 × wash buffer to each well without inverting by placing the plate on the vacuum manifold and applying vacuum. Detection antibodies were then added and shake the plates @500 rpm for 2 h at room temperature. Added SA-PE reagent and sealed, then shake the plates @500 rpm for 30 min. Washed and vacuumed then re-suspend the beads on a plate shaker for 1 min. Samples were transferred from the filter plate to the FACS plate to read samples on a flow cytometer. The flow cytometer and acquisition software setting were established according to the manufacturer's instructions. Purified anti-human CD314 (NKG2D) blocking antibody (clone 1D11) was used as control.

### Western blot analysis

Cells were seeded at 1 × 10^5^/ml onto 60 mm dishes, treated, collected, lysed, and immunoblotted with indicated antibody. The detailed procedure was described in our previous publications [52, 56]. Briefly, cell lysis was completed by sonication and the total protein was separated on an SDS-PAGE, transferred onto a PVDF membrane (Millipore), and immunoblotted with indicated primary antibody. Antibodies to PD-L1 (cat no. A19135) and to NKG2D (cat no. A6123) were purchased from Abclonal. Antibodies to IDO1 (cat no. 51851) and (cat no. 86630) were purchased from Cell Signaling Technology. Antibody to TDO2 (cat no. NBP2-45,995) was purchased from Novus Biologicals. Antibody to IDO2 (cat no.703150) was purchased from ThermoFisher Scientific. All antibody dilutions were at 1:1000, except for β-Actin (Sigma, cat no. A1978) which was diluted at 1:10,000. Bands were measured using a molecular imager Chemidoc system with Quality One software (Bio-Rad Laboratories).

### Immunohistochemistry staining

Immunohistochemical staining was performed according to our previous method with some modifications [5]. The tissue slides (4 µm of tissue slide) were placed in an incubator at 55° to melt the paraffin-embedded sections, rehydrated by immersing it in xylene followed by 100% 95% 70% 50% ethanol, and rinsed with deionized H_2_O. Next, a 0.3% hydrogen peroxidase blocking agent was applied to block any internal peroxidase activity. A target retrieval solution (citric buffer; pH 6.0) was then used to enhance the staining. Samples were incubated overnight with Anti-IDO-1 Antibody (Santa Cruz cat no. SC137012) primary antibodies at 1:250 in antibody dilution solution (Dako, Santa Clara, CA, USA). Using Mouse and Rabbit Specific HRP/DAB (ABC) detection IHC kit (Abcam cat# 64,264), secondary antibody biotinylated solution was added for 15 min, washed, and streptavidin conjugated to peroxidase (HRP) solution was added for 15 min. DAB chromogen was then applied to the slides to reveal antibody staining and then counter-stained with hematoxylin. Following the counterstain, the tissue slides were rinsed in tap water and placed in 50% ethanol followed by 70%95%100% ethanol, and xylene. A cover slip was then placed over each slide, secured using a mounting solution, and stored at room temperature. Staining results were read and captured by Keyence All-in-One Microscope BZ-X710 equipped with hybrid cell count image quantification software.

### Statistical analysis

Data from in vitro experiments were performed from three separate biological replicates which were isolated and analyzed in technical triplicates. Separate measurements using the two-tailed *t* test and the results were expressed as mean ± standard deviation. One-way ANOVA was done for the comparison of data of different groups followed by post hoc analysis. Tukey’s multiple comparisons post hoc analysis was used when comparing the mean of each column with the mean of every other column. Dunnett’s multiple comparisons post hoc analysis was used when comparing the mean of each column with the mean of a control column. The level of significance was at *P < 0.05, **P < 0.005. ***P < 0.0005, ****P < *0.0001.*

## Supplementary Information

**Additional file 1:** **Supplementary Figure S1A.** **Figure S1A.** Gating strategy used to identify NK and CD8+T cells;viable cells were gated based on FSC-A versus Fixable Viability Dye;cells were gated on CD45+ and further divided into CD3- and CD3+ fractions;CD56+ and NKG2D+ cells were selected from the CD3- fraction;CD8+ and NKG2D+ cells were selected from CD3+ fraction. Gating strategy used to identify human-regulator T cells.Viable cells were gated based on FSC-A versus Fixable Viability Dye.Cells were gated on CD45+CD127low and CD4+CD25+ were gated.CD4+ and intracellular FoxP3+ were selected from CD4+CD25+ population. Gating strategy used to identify human-MDSC cells.Viable cells were gated based on FSC-A versus Fixable Viability Dye.Cells were gated on CD45+ and CD14-and HLA-DR^low^ were gated.CD11b+ and CD33+ were selected from the CD14-HLA-DR^low^ population. Note: Fluorescence minus onewas used to identify gating boundaries. **Supplementary** **Figure S1B.** Gating strategy used to identify mouse-regulator T cells.Viable cells were gated based on CD45+ versus Fixable Viability Dye.CD4+CD25+ were gated from CD45+live.CD4+ and intracellular FoxP3+ cells were selected from CD4+CD25+ population. Gating strategy used to identify mouse-CD8+ T cells.Viable cells were gated based on CD45+ versus Fixable Viability Dye.CD3+CD8+ were gated from CD45+live.NKG2D+and CD8+ cells were selected from CD3+CD8+population. Gating strategy used to identify mouse-NK-cells.Viable cells were gated based on CD45+ versus Fixable Viability Dye.CD3-CD11b+ were gated from CD45+live.NKG2D+ and CD49b+ cells were selected from CD3-CD11b+population. Gating strategy used to identify mouse-MDSC cells.Viable cells were gated based on CD45+ versus Fixable Viability Dye.F4/80^low^ and Ly6C+were gated from CD45+live.CD11b+ and Gr1+ were selected from the F4/80^low^ and Ly6C+population. Note: Fluorescence minus onewas used to identify gating boundaries. **Figure S2.** IDO-mediated KYN production from CR cells suppressed immunomodulatory profile.Immunoblot of IDO1, IDO2, and TDO2. Resistant cells were treated with either IDO1 inhibitor or shRNA targeting IDO1.Detection of amino acid KYN and TRP in culture supernatants of F vs FC. The concentration of amino acids is measured using Amino Acid Analyzer Biochrome30+.Immune profile assayed by flow cytometry. Using the same experimental co-condition as Fig. 1C, IDO1 inhibition significantly enhanced percent of NKG2D on NK cellsand percent of NKG2D on CD8+cells, but significantly suppressed Tregand MDSCfrequencies in CD45+ lymphocytes.The indicated cytokines are quantified in culture supernatants by LEGENDplex™ bead-based immunoassay. The panel below indicated that anti-NKG2D blocking antibodies blunt the effect of IDO1 inhibition. *Note:* To detect the MDSC population, cells were activated by PHA+IL2 instead of antiCD2/28+IL2. In all the experiments, data presented as mean ± SEM of 3 independent experiments and were analyzed using one-way ANOVA followed by Tukey with *P<0.05, **P<0.005, ***P<0.0005, ****P<0.0001. **Figure S3.** Effect of inhibitors on the expression of IDO1/2 and TDO2 in the sensitive and resistant tumors.Expression of IDO2 was detected via western blot analysis in LLC and LLC-CR tumors from syngeneic mice treated with a control vehicle, IDO1 inhibitor, a dual inhibitor for IDO1/TDO2.IDO1 expression. Relative mRNA levels of IDO1 in LLC-CR vs. LLC-CR transfected with *CRISPR-IDO1* knockout clone SG1 and SG2 Actin was used as an internal control. The results shown in the graph were calculated with the ΔΔCt method by setting the mRNA level of LLC-CR as 1. IHC of IDO1 expression indicated complete IDO1 knockout in vivo. Box graph indicated quantification of IHCright panel.Expression of IDO1, IDO2, and TDO2 was detected via western blot analysis *in vivo*. LLC and LLC-CR tumors from syngeneic mice treated with control vehicleor TDO1 inhibitor. The right panel indicated the protein quantification of IDO1.Serum KYN and TRP levels in mice treated with LM10. Data were analyzed using one-way ANOVA followed by Dunnett’s multiple comparisons with *P<0.05, **P<0.005, ***P<0.0005, ****P<0.0001. **Figure S4.** Inhibition of IDO1 and TDO2 further suppressed KYN production and CR cell growth.The amino acid KYN and TRP concentrations were detected in human cell cultures. Blocking both IDO1 and TDO2 led to a significant reduction in KYN.Growth inhibitory assay.Cells were treated with indicated drugs for 72h. Intracellular KYN accumulation was detected by the amino acid analyzer. Note that IC50 concentrations of CR cells were used.Colony formations of human and mouse cell lines were determined in CSvs. CRcells treated with 25 µM of AT-0174 or epacadostat for 3 days and reseeded for 12 more days. Data were analyzed using one-way ANOVA followed by Tukey’s multiple comparison analysis with *P<0.05, **P<0.005, ***P<0.0005, ****P<0.0001. **Figure S5.** Antitumor efficacy of dual inhibitor+PD-1 blockage. Antitumor efficacy of AT-0174 in combination with anti-PD-1 antibody in syngeneic mice bearing CS tumor, LLC. No significant results of tumor growth and weight were found in the combination treatment. **Supplementary Table S1.** IDO1 and TDO2 inhibitory activities and chemical formula of AT-0174. **Supplementary Table S2.** Antibodies for flow cytometry experiments. **Supplementary Table S3.** shRNA constructs in lentiviral GFP vector. **Supplementary Table S4.** CRISPR/Cas9-Directed Genome Editing of Ido1.

## Data Availability

All data that support the findings of this study are included in the article and its supporting information. A copy of all data analyzed is available from the corresponding author upon request. The original contributions presented in the study are included in the article/Supplementary material, further inquiries can be directed to the corresponding author.
