# Supplementary material for: Dual inhibition of IDO1/TDO2 enhances anti-tumor immunity in platinum-resistant non-small cell lung cancer
Source: Cancer Metab. 2023 May 24;11:7. doi: 10.1186/s40170-023-00307-1 (PMC10207715; doi:10.1186/s40170-023-00307-1)
Supplement: Supplementary file 1 — Additional file 1: Supplementary Figure S1A. Figure S1A. Gating strategy used to identify NK and CD8+T cells;viable cells were gated based on FSC-A versus Fixable Viability Dye;cells were gated on CD45+ and further divided into CD3- and CD3+ fractions;CD56+ and NKG2D+ cells were selected from the CD3- fraction;CD8+ and NKG2D+ cells were selected from CD3+ fraction. Gating strategy used to identify human-regulator T cells.Viable cells were gated based on FSC-A versus Fixable Viability Dye.Cells were gated on CD45+CD127low and CD4+CD25+ were gated.CD4+ and intracellular FoxP3+ were selected from CD4+CD25+ population. Gating strategy used to identify human-MDSC cells.Viable cells were gated based on FSC-A versus Fixable Viability Dye.Cells were gated on CD45+ and CD14-and HLA-DRlow were gated.CD11b+ and CD33+ were selected from the CD14-HLA-DRlow population. Note: Fluorescence minus onewas used to identify gating boundaries. Supplementary Figure S1B. Gating strategy used to identify mouse-regulator T cells.Viable cells were gated based on CD45+ versus Fixable Viability Dye.CD4+CD25+ were gated from CD45+live.CD4+ and intracellular FoxP3+ cells were selected from CD4+CD25+ population. Gating strategy used to identify mouse-CD8+ T cells.Viable cells were gated based on CD45+ versus Fixable Viability Dye.CD3+CD8+ were gated from CD45+live.NKG2D+and CD8+ cells were selected from CD3+CD8+population. Gating strategy used to identify mouse-NK-cells.Viable cells were gated based on CD45+ versus Fixable Viability Dye.CD3-CD11b+ were gated from CD45+live.NKG2D+ and CD49b+ cells were selected from CD3-CD11b+population. Gating strategy used to identify mouse-MDSC cells.Viable cells were gated based on CD45+ versus Fixable Viability Dye.F4/80low and Ly6C+were gated from CD45+live.CD11b+ and Gr1+ were selected from the F4/80low and Ly6C+population. Note: Fluorescence minus onewas used to identify gating boundaries. Figure S2. IDO-mediated KYN production from CR cells suppressed immu [file 40170_2023_307_MOESM1_ESM.pptx]

## Slide 1
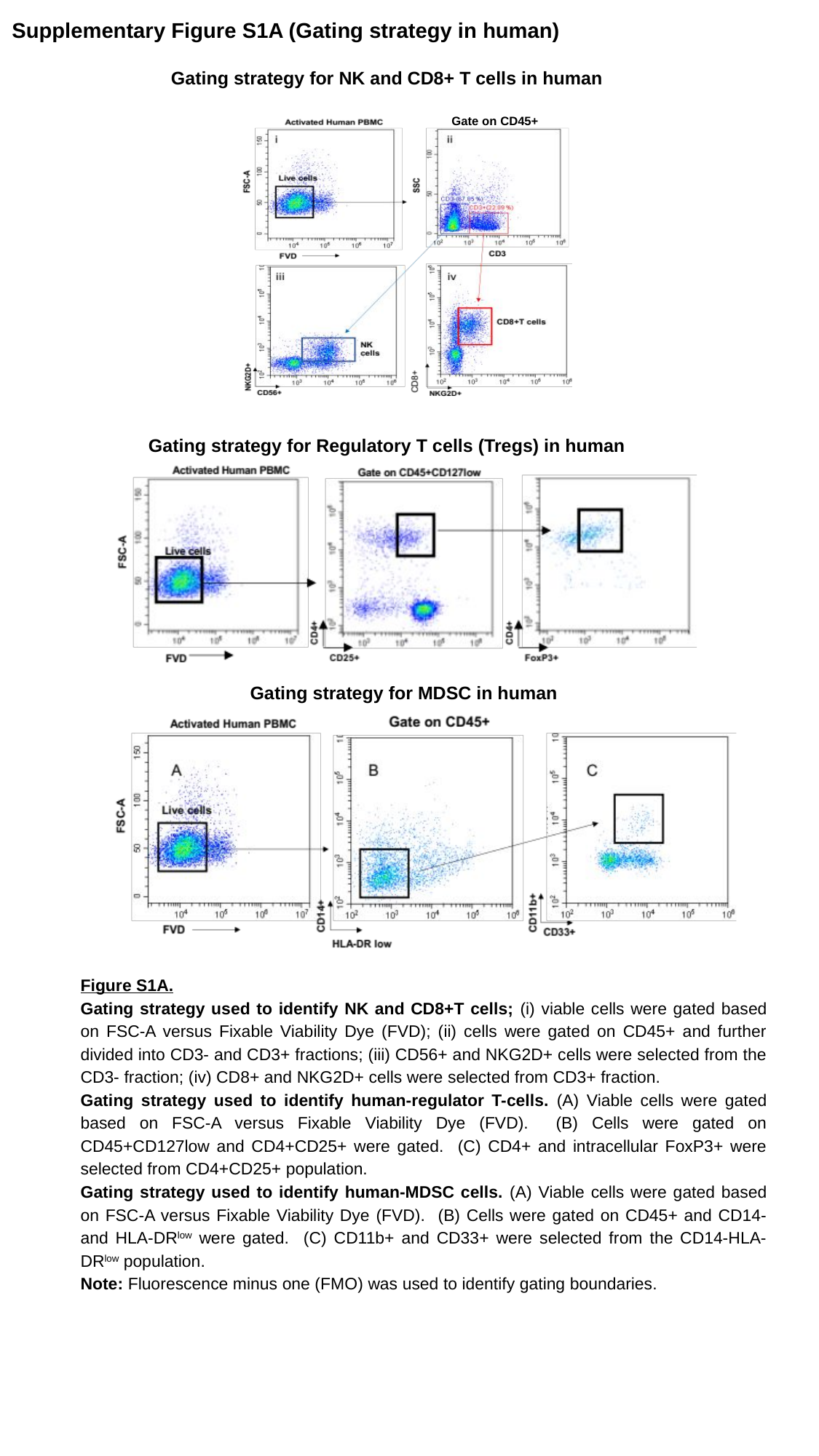

Supplementary Figure S1A (Gating strategy in human)
Gating strategy for NK and CD8+ T cells in human
Gate on CD45+
Gating strategy for Regulatory T cells (Tregs) in human
Gating strategy for MDSC in human
Figure S1A.
Gating strategy used to identify NK and CD8+T cells; (i) viable cells were gated based on FSC-A versus Fixable Viability Dye (FVD); (ii) cells were gated on CD45+ and further divided into CD3- and CD3+ fractions; (iii) CD56+ and NKG2D+ cells were selected from the CD3- fraction; (iv) CD8+ and NKG2D+ cells were selected from CD3+ fraction.
Gating strategy used to identify human-regulator T-cells. (A) Viable cells were gated based on FSC-A versus Fixable Viability Dye (FVD). (B) Cells were gated on CD45+CD127low and CD4+CD25+ were gated. (C) CD4+ and intracellular FoxP3+ were selected from CD4+CD25+ population.
Gating strategy used to identify human-MDSC cells. (A) Viable cells were gated based on FSC-A versus Fixable Viability Dye (FVD). (B) Cells were gated on CD45+ and CD14-and HLA-DRlow were gated. (C) CD11b+ and CD33+ were selected from the CD14-HLA-DRlow population.
Note: Fluorescence minus one (FMO) was used to identify gating boundaries.

## Slide 2
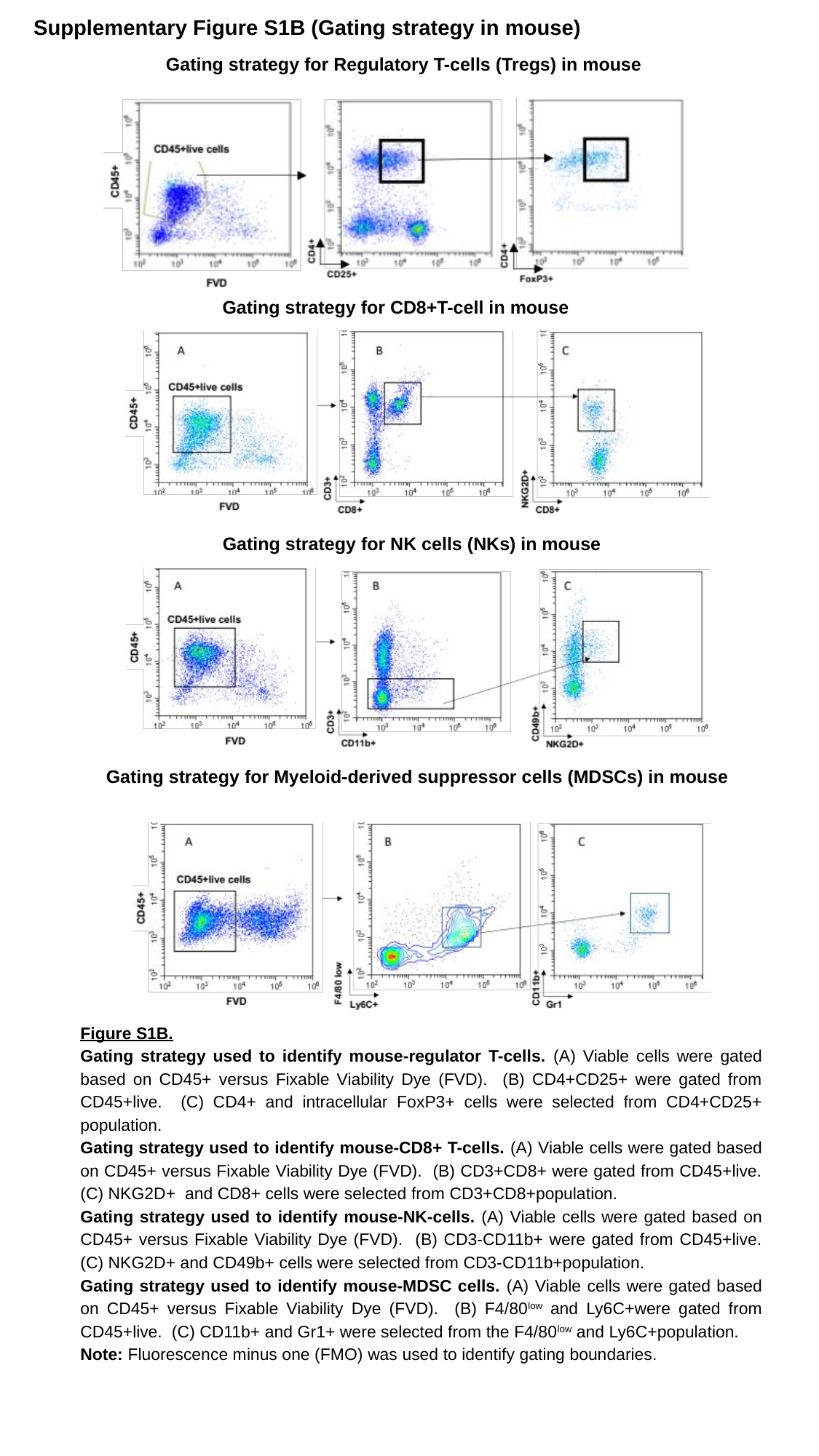

Supplementary Figure S1B (Gating strategy in mouse)
Gating strategy for Regulatory T-cells (Tregs) in mouse
Gating strategy for CD8+T-cell in mouse
Gating strategy for NK cells (NKs) in mouse
Gating strategy for Myeloid-derived suppressor cells (MDSCs) in mouse
Figure S1B.
Gating strategy used to identify mouse-regulator T-cells. (A) Viable cells were gated based on CD45+ versus Fixable Viability Dye (FVD). (B) CD4+CD25+ were gated from CD45+live. (C) CD4+ and intracellular FoxP3+ cells were selected from CD4+CD25+ population.
Gating strategy used to identify mouse-CD8+ T-cells. (A) Viable cells were gated based on CD45+ versus Fixable Viability Dye (FVD). (B) CD3+CD8+ were gated from CD45+live. (C) NKG2D+ and CD8+ cells were selected from CD3+CD8+population.
Gating strategy used to identify mouse-NK-cells. (A) Viable cells were gated based on CD45+ versus Fixable Viability Dye (FVD). (B) CD3-CD11b+ were gated from CD45+live. (C) NKG2D+ and CD49b+ cells were selected from CD3-CD11b+population.
Gating strategy used to identify mouse-MDSC cells. (A) Viable cells were gated based on CD45+ versus Fixable Viability Dye (FVD). (B) F4/80low and Ly6C+were gated from CD45+live. (C) CD11b+ and Gr1+ were selected from the F4/80low and Ly6C+population.
Note: Fluorescence minus one (FMO) was used to identify gating boundaries.

## Slide 3
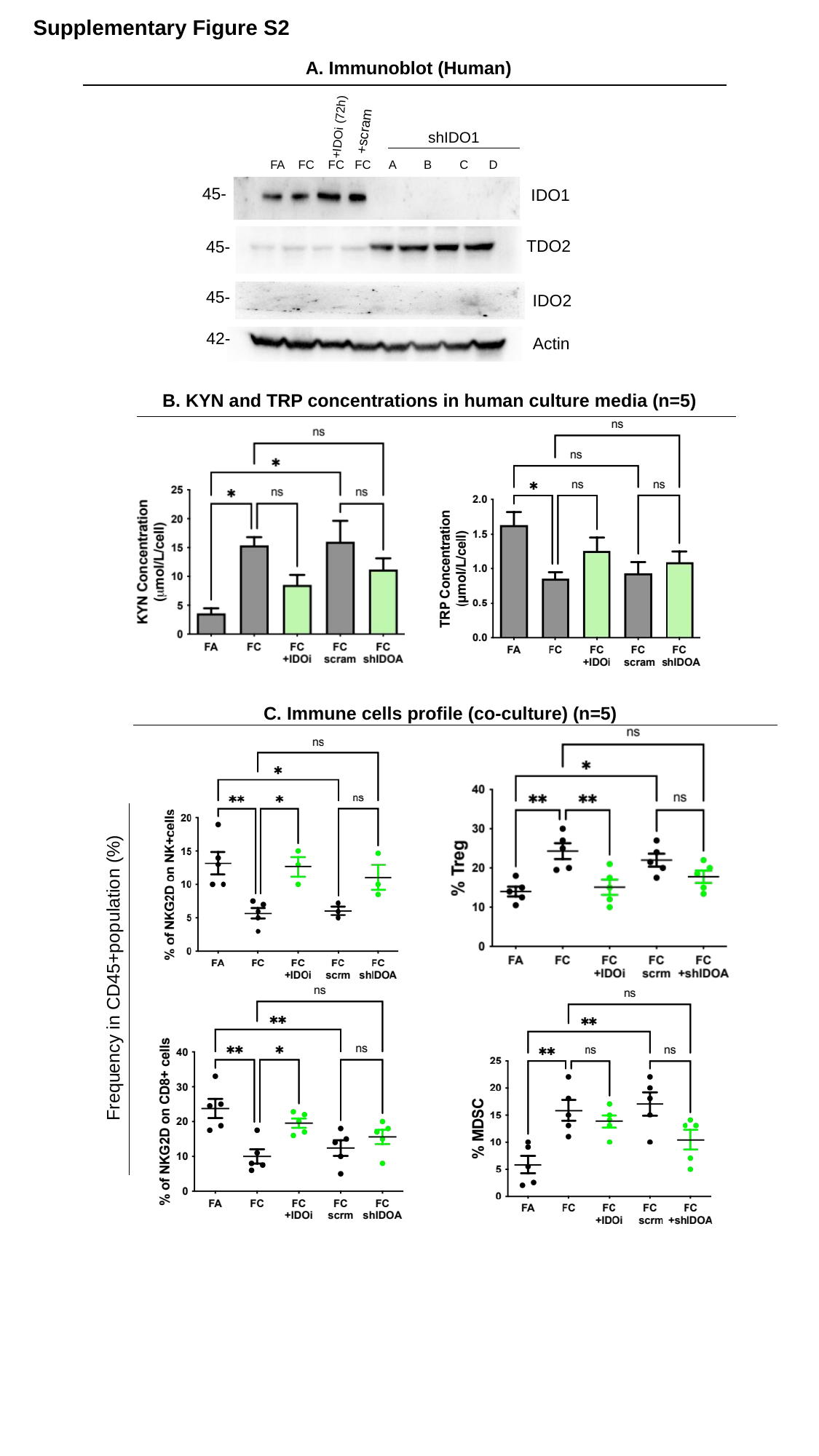

Supplementary Figure S2
A. Immunoblot (Human)
+IDOi (72h)
+scram
shIDO1
A B C D
 FA FC FC FC
45-
IDO1
TDO2
45-
45-
IDO2
42-
Actin
B. KYN and TRP concentrations in human culture media (n=5)
C. Immune cells profile (co-culture) (n=5)
Frequency in CD45+population (%)

## Slide 4
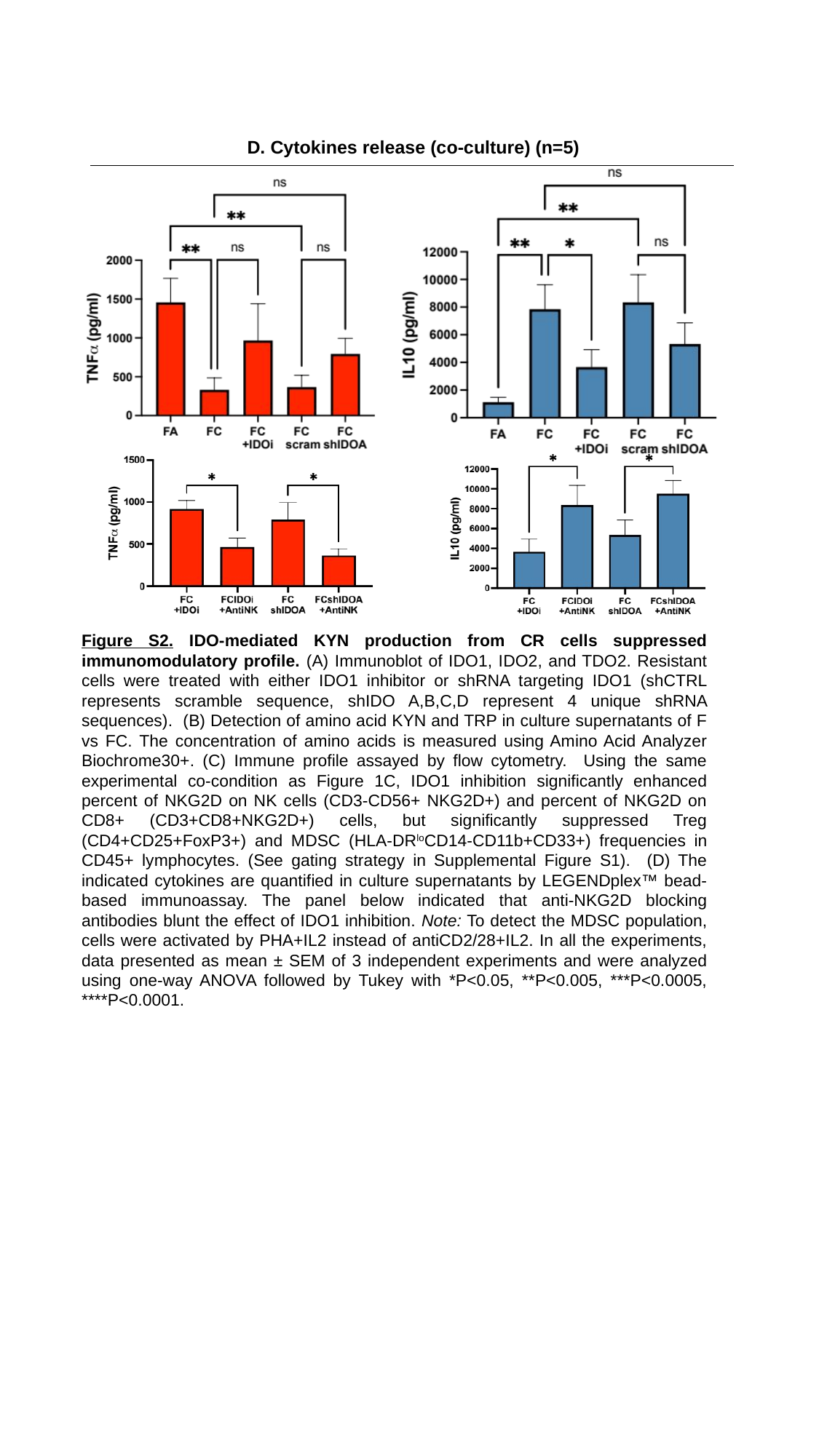

D. Cytokines release (co-culture) (n=5)
Figure S2. IDO-mediated KYN production from CR cells suppressed immunomodulatory profile. (A) Immunoblot of IDO1, IDO2, and TDO2. Resistant cells were treated with either IDO1 inhibitor or shRNA targeting IDO1 (shCTRL represents scramble sequence, shIDO A,B,C,D represent 4 unique shRNA sequences). (B) Detection of amino acid KYN and TRP in culture supernatants of F vs FC. The concentration of amino acids is measured using Amino Acid Analyzer Biochrome30+. (C) Immune profile assayed by flow cytometry. Using the same experimental co-condition as Figure 1C, IDO1 inhibition significantly enhanced percent of NKG2D on NK cells (CD3-CD56+ NKG2D+) and percent of NKG2D on CD8+ (CD3+CD8+NKG2D+) cells, but significantly suppressed Treg (CD4+CD25+FoxP3+) and MDSC (HLA-DRloCD14-CD11b+CD33+) frequencies in CD45+ lymphocytes. (See gating strategy in Supplemental Figure S1). (D) The indicated cytokines are quantified in culture supernatants by LEGENDplex™ bead-based immunoassay. The panel below indicated that anti-NKG2D blocking antibodies blunt the effect of IDO1 inhibition. Note: To detect the MDSC population, cells were activated by PHA+IL2 instead of antiCD2/28+IL2. In all the experiments, data presented as mean ± SEM of 3 independent experiments and were analyzed using one-way ANOVA followed by Tukey with *P<0.05, **P<0.005, ***P<0.0005, ****P<0.0001.

## Slide 5
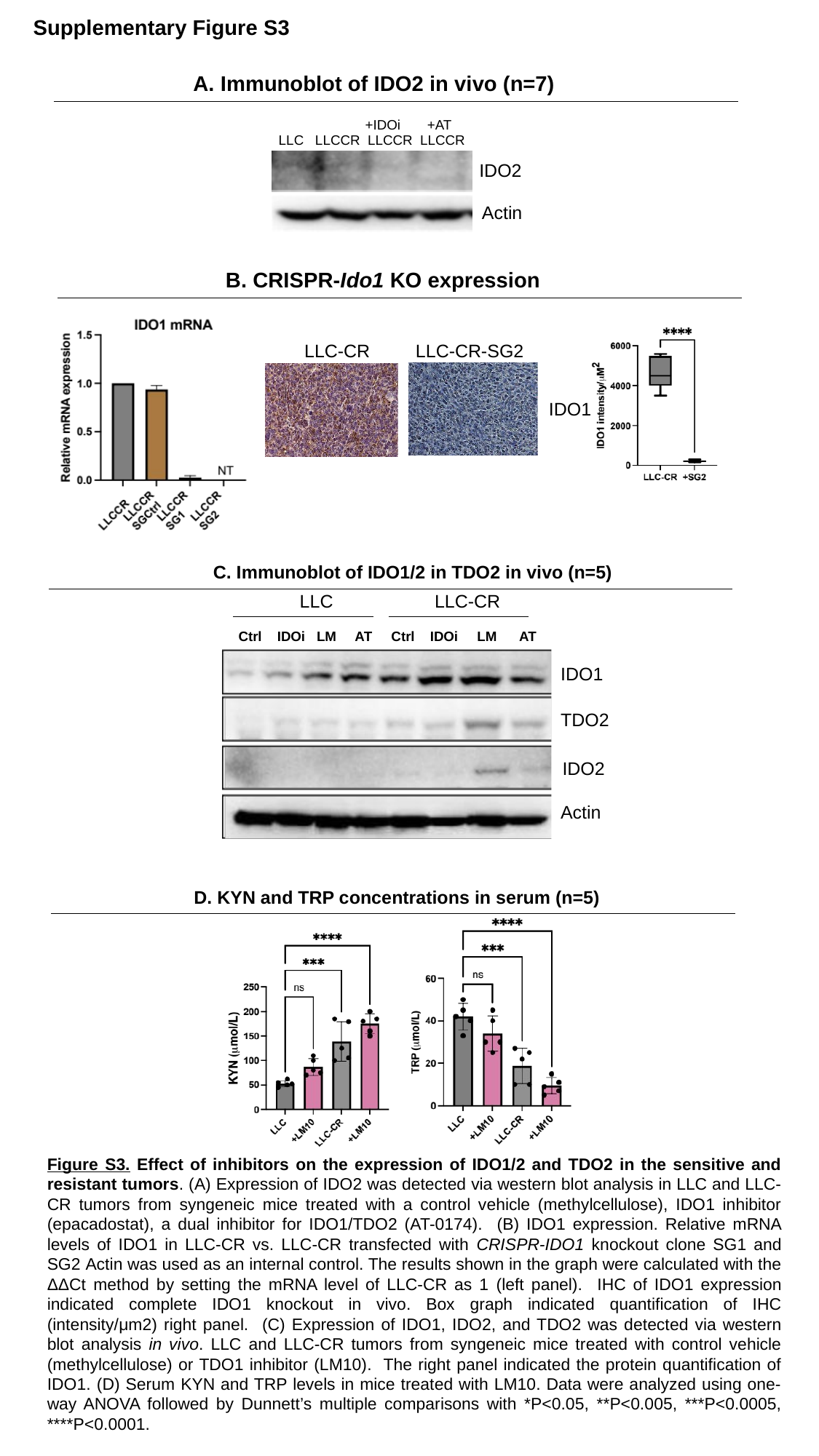

Supplementary Figure S3
A. Immunoblot of IDO2 in vivo (n=7)
+IDOi +AT
LLC LLCCR LLCCR LLCCR
IDO2
Actin
B. CRISPR-Ido1 KO expression
LLC-CR LLC-CR-SG2
IDO1
C. Immunoblot of IDO1/2 in TDO2 in vivo (n=5)
LLC LLC-CR
Ctrl IDOi LM AT Ctrl IDOi LM AT
IDO1
TDO2
IDO2
Actin
D. KYN and TRP concentrations in serum (n=5)
Figure S3. Effect of inhibitors on the expression of IDO1/2 and TDO2 in the sensitive and resistant tumors. (A) Expression of IDO2 was detected via western blot analysis in LLC and LLC-CR tumors from syngeneic mice treated with a control vehicle (methylcellulose), IDO1 inhibitor (epacadostat), a dual inhibitor for IDO1/TDO2 (AT-0174). (B) IDO1 expression. Relative mRNA levels of IDO1 in LLC-CR vs. LLC-CR transfected with CRISPR-IDO1 knockout clone SG1 and SG2 Actin was used as an internal control. The results shown in the graph were calculated with the ΔΔCt method by setting the mRNA level of LLC-CR as 1 (left panel). IHC of IDO1 expression indicated complete IDO1 knockout in vivo. Box graph indicated quantification of IHC (intensity/μm2) right panel. (C) Expression of IDO1, IDO2, and TDO2 was detected via western blot analysis in vivo. LLC and LLC-CR tumors from syngeneic mice treated with control vehicle (methylcellulose) or TDO1 inhibitor (LM10). The right panel indicated the protein quantification of IDO1. (D) Serum KYN and TRP levels in mice treated with LM10. Data were analyzed using one-way ANOVA followed by Dunnett’s multiple comparisons with *P<0.05, **P<0.005, ***P<0.0005, ****P<0.0001.

## Slide 6
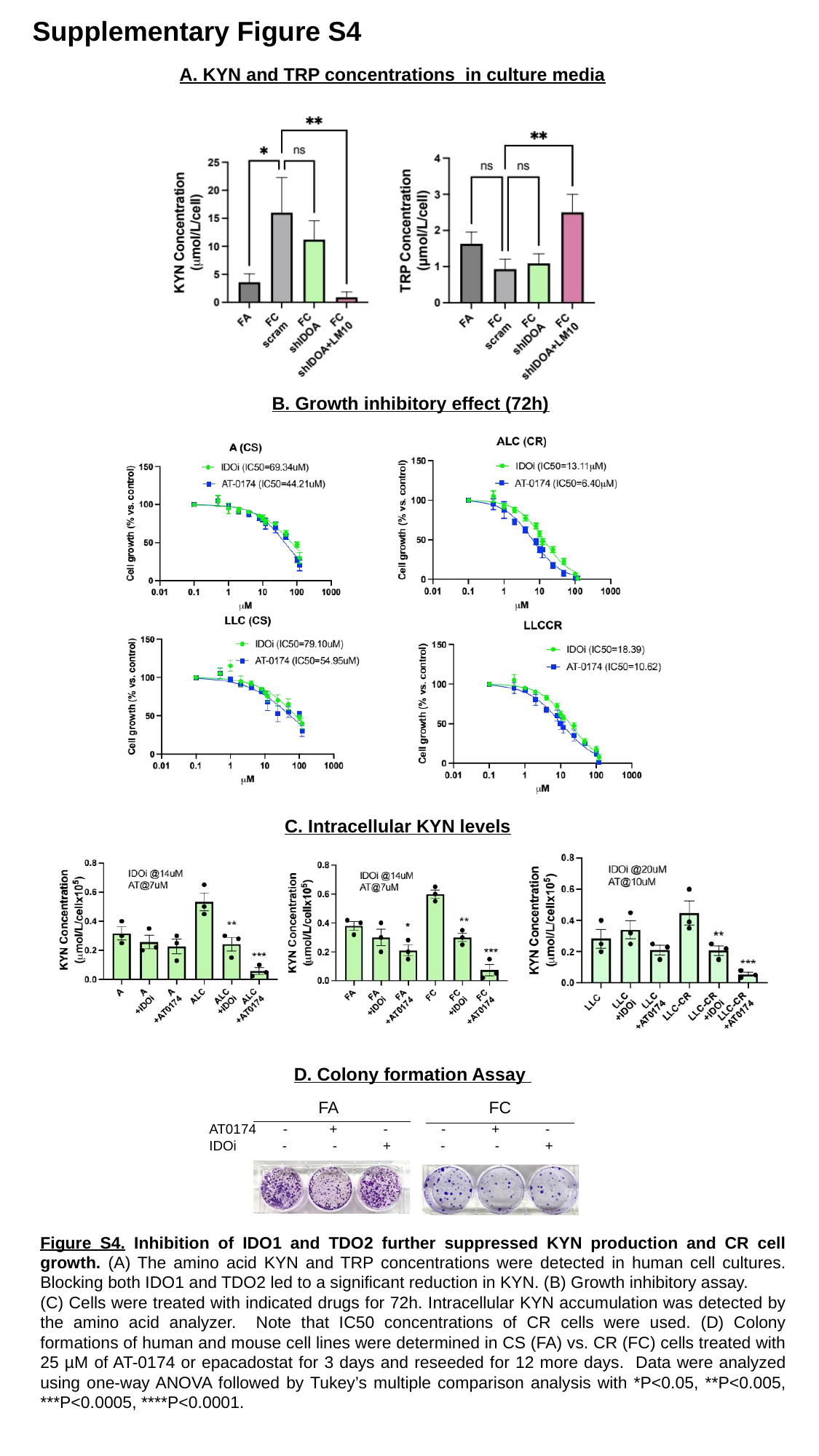

Supplementary Figure S4
A. KYN and TRP concentrations in culture media
B. Growth inhibitory effect (72h)
C. Intracellular KYN levels
D. Colony formation Assay
FA FC
AT0174 - + - - + -
IDOi - - + - - +
Figure S4. Inhibition of IDO1 and TDO2 further suppressed KYN production and CR cell growth. (A) The amino acid KYN and TRP concentrations were detected in human cell cultures. Blocking both IDO1 and TDO2 led to a significant reduction in KYN. (B) Growth inhibitory assay.
(C) Cells were treated with indicated drugs for 72h. Intracellular KYN accumulation was detected by the amino acid analyzer. Note that IC50 concentrations of CR cells were used. (D) Colony formations of human and mouse cell lines were determined in CS (FA) vs. CR (FC) cells treated with 25 µM of AT-0174 or epacadostat for 3 days and reseeded for 12 more days. Data were analyzed using one-way ANOVA followed by Tukey’s multiple comparison analysis with *P<0.05, **P<0.005, ***P<0.0005, ****P<0.0001.

## Slide 7
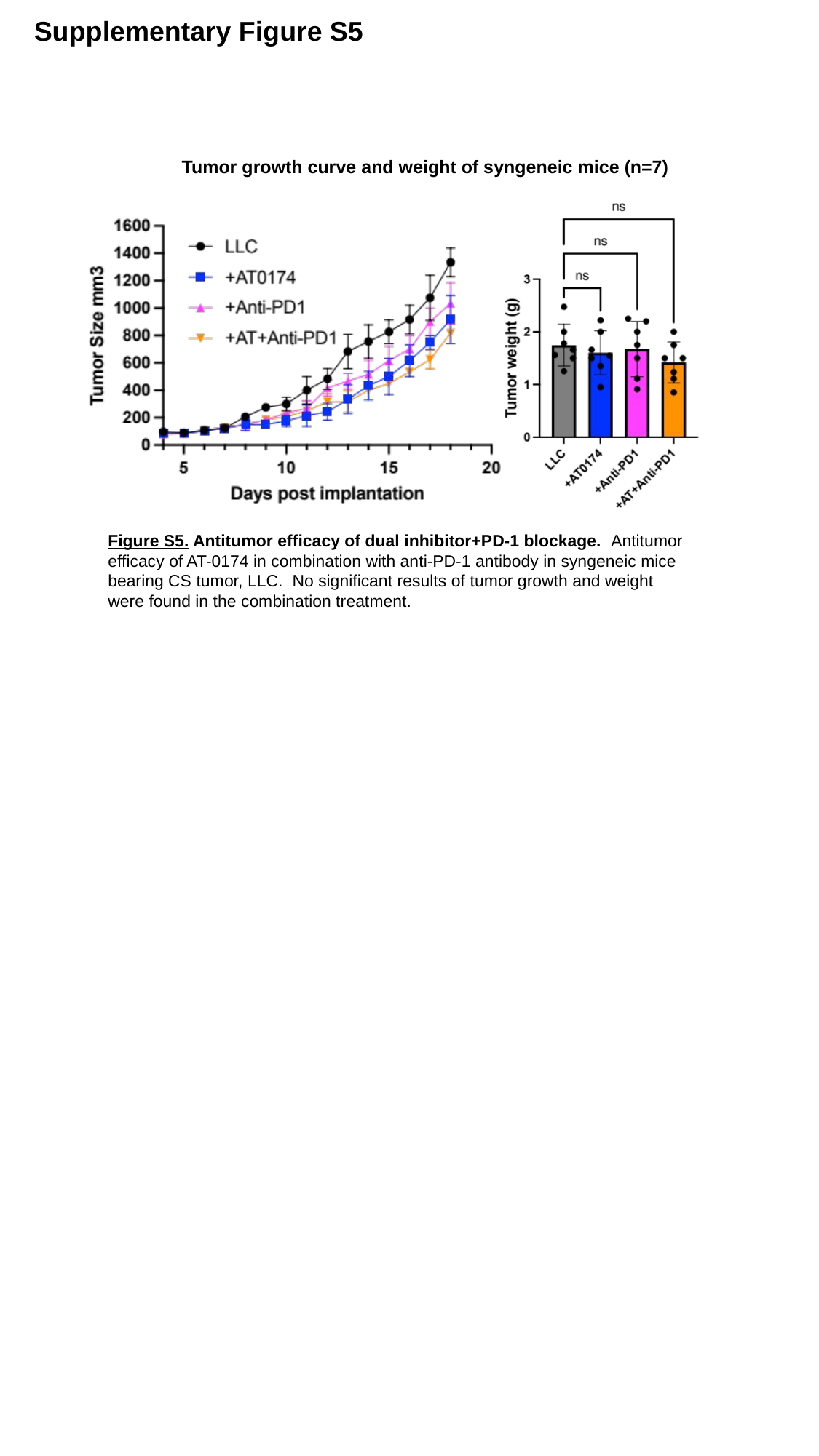

Supplementary Figure S5
Tumor growth curve and weight of syngeneic mice (n=7)
Figure S5. Antitumor efficacy of dual inhibitor+PD-1 blockage. Antitumor efficacy of AT-0174 in combination with anti-PD-1 antibody in syngeneic mice bearing CS tumor, LLC. No significant results of tumor growth and weight were found in the combination treatment.

## Slide 8
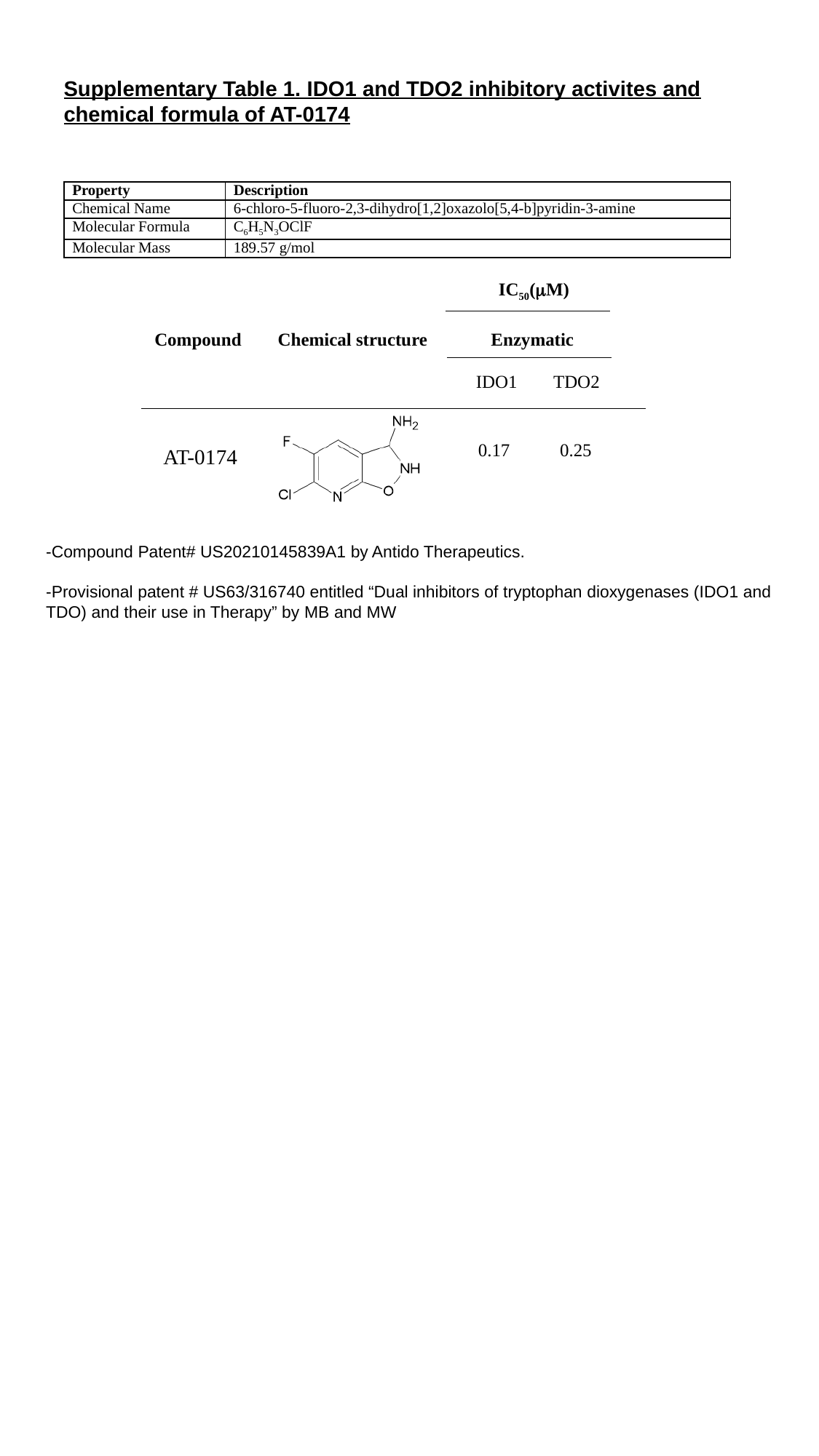

Supplementary Table 1. IDO1 and TDO2 inhibitory activites and chemical formula of AT-0174
| Property | Description |
| --- | --- |
| Chemical Name | 6-chloro-5-fluoro-2,3-dihydro[1,2]oxazolo[5,4-b]pyridin-3-amine |
| Molecular Formula | C6H5N3OClF |
| Molecular Mass | 189.57 g/mol |
IC50(mM)
Compound Chemical structure Enzymatic
IDO1 TDO2
0.17 0.25
AT-0174
-Compound Patent# US20210145839A1 by Antido Therapeutics.
-Provisional patent # US63/316740 entitled “Dual inhibitors of tryptophan dioxygenases (IDO1 and TDO) and their use in Therapy” by MB and MW

## Slide 9
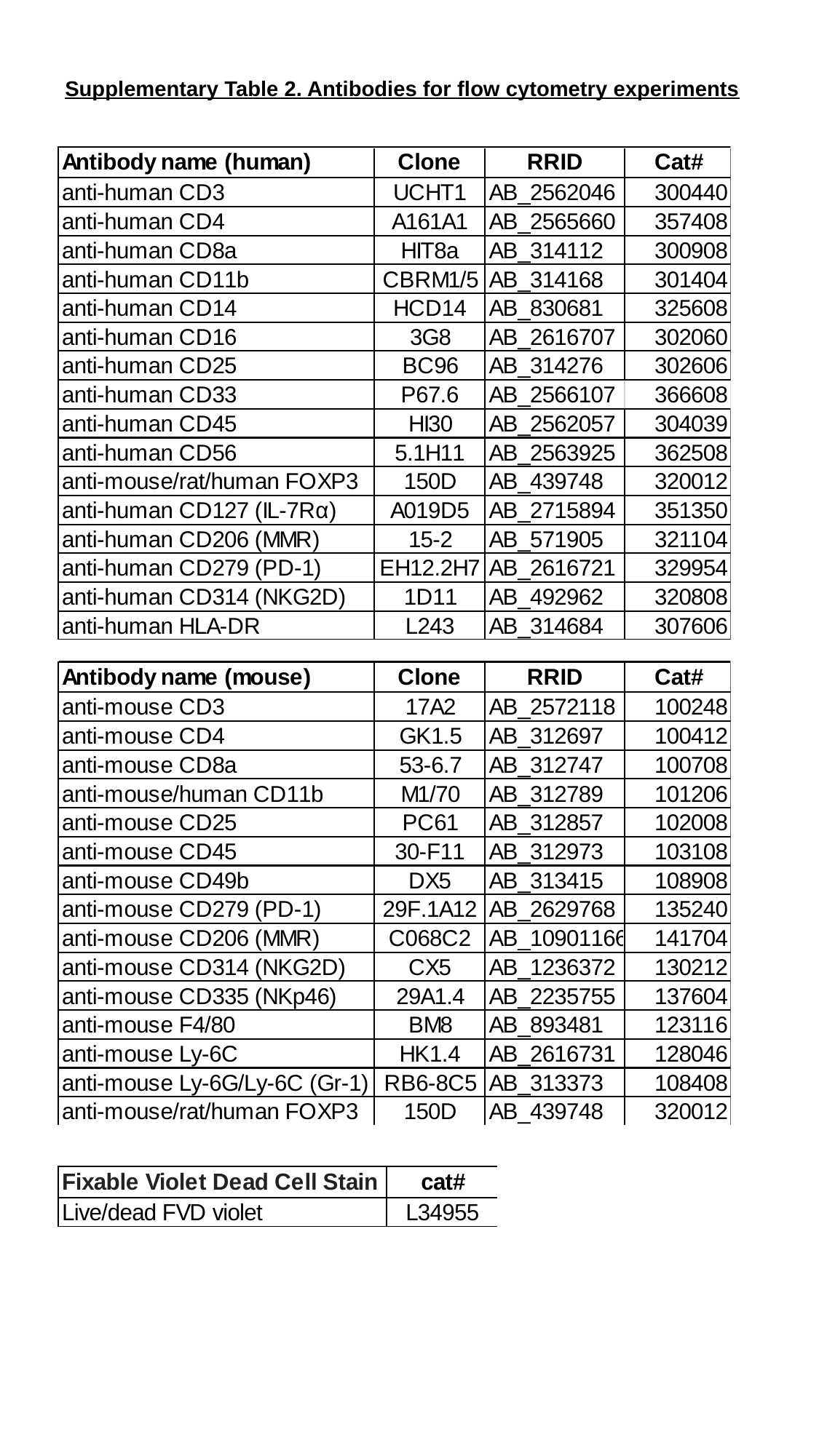

Supplementary Table 2. Antibodies for flow cytometry experiments

## Slide 10
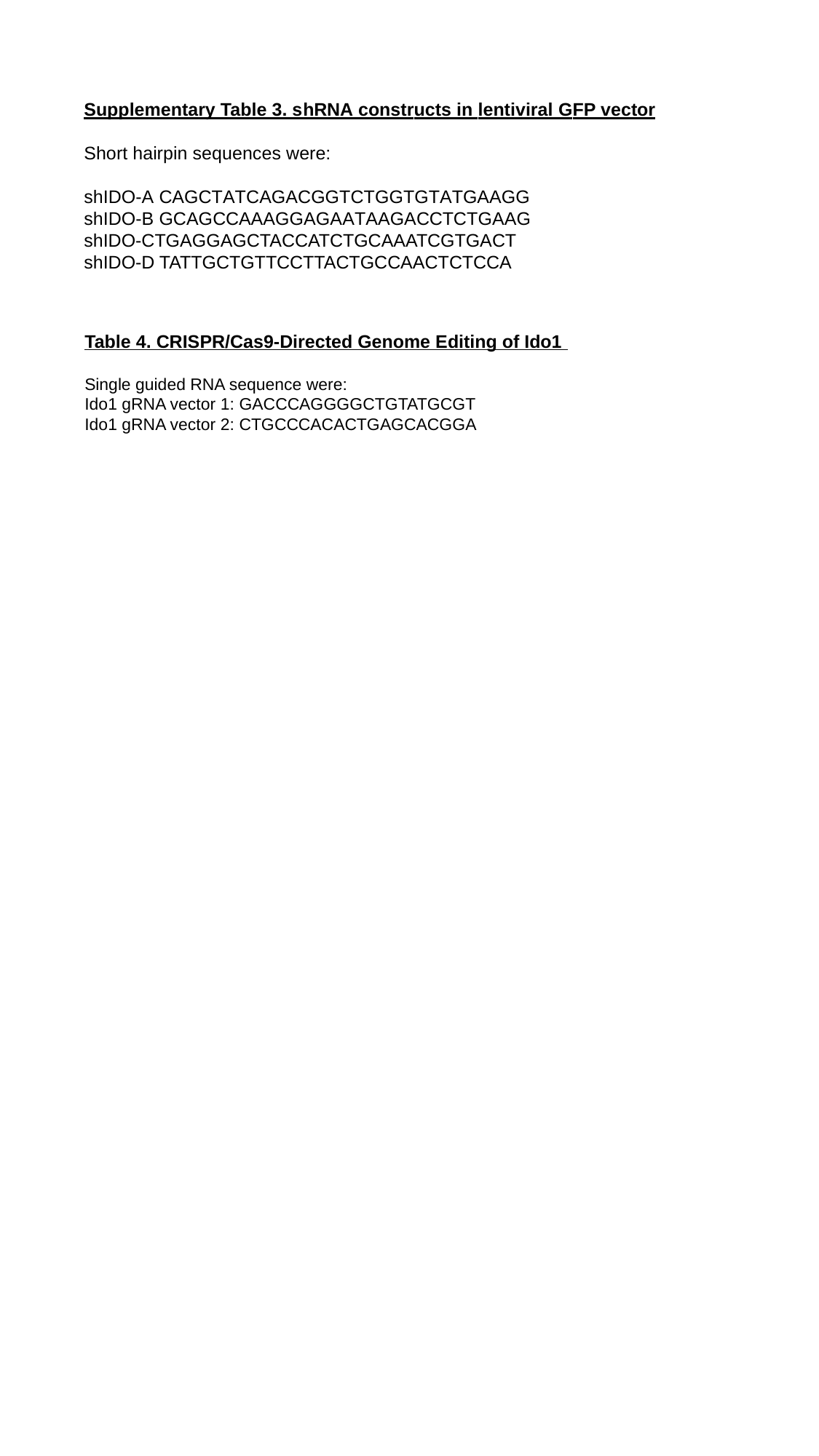

Supplementary Table 3. shRNA constructs in lentiviral GFP vector
Short hairpin sequences were:
shIDO-A CAGCTATCAGACGGTCTGGTGTATGAAGG
shIDO-B GCAGCCAAAGGAGAATAAGACCTCTGAAG
shIDO-CTGAGGAGCTACCATCTGCAAATCGTGACT
shIDO-D TATTGCTGTTCCTTACTGCCAACTCTCCA
Table 4. CRISPR/Cas9-Directed Genome Editing of Ido1
Single guided RNA sequence were:
Ido1 gRNA vector 1: GACCCAGGGGCTGTATGCGT
Ido1 gRNA vector 2: CTGCCCACACTGAGCACGGA
